# Supplementary material for: Communication pathways bridge local and global conformations in an IgG4 antibody
Source: Sci Rep. 2021 Dec 1;11:23197. doi: 10.1038/s41598-021-02323-x (PMC8636491; doi:10.1038/s41598-021-02323-x)
Supplement: Supplementary file 1 — Supplementary Information 1. [file 41598_2021_2323_MOESM1_ESM.pdf]

Supporting Information for: Communication Pathways Bridge Local and Global Conformations in an IgG4 Antibody

Thomas Tarenzi*[1,2], Marta Rigoli*[1,2], Raffaello Potestio[1,2]

[1] *Physics Department, University of Trento, Via Sommarive 14, Povo (TN), Italy*

[2] *INFN-TIFPA, Trento Institute for Fundamental Physics and Applications, Via Sommarive 14, Povo (TN), Italy*

* Contributed equally to this work

S1 Methods

Principal component analysis In order to tell whether two structural domains $\mathcal{A}$ and $\mathcal{B}$ move in the same direction in a cluster, we computed the following quantity for each of the first three principal components:

$$q_k^{AB} = \frac{1}{NM} \sum_{i \in \mathcal{A}} \sum_{j \in \mathcal{B}} \mathbf{v}_i \cdot \mathbf{u}_j \tag{1}$$

Here, $\mathbf{v}_i$ and $\mathbf{u}_j$ are vectors of the k-th mode, relative to the residues i and j, and N and M are the numbers of residues in domains $\mathcal{A}$ and $\mathcal{B}$, respectively. Results for each mode are combined, and weighted by their corresponding eigenvalues λ_k:

$$Q^{AB} = \frac{\sum_{k=1}^{3} q_k^{AB} \lambda_k}{\sum_{k=1}^{3} \lambda_k} \tag{2}$$

Each entry Q^{AB} in the resulting matrix refers to a couple of structural domains. Positive values correspond to parallel movements, while negative values to motions in an antiparallel direction.

MM/PBSA calculations In the MM/PBSA approach [1], the binding free energy ΔG_{bind} between the antibody (Ab) and the antigen (Ag), namely

$$\Delta G_{bind} = G_{complex} - G_{Ab} - G_{Ag} \tag{3}$$

is written as a sum of different contribution:

$$\Delta G_{bind} = \Delta H - T\Delta S = \Delta E_{MM} + \Delta G_{sol} - T\Delta S \quad (4)$$

Here, ΔE_{MM}, ΔG_{sol} and ΔS are the changes in the gas-phase molecular mechanics energy, solvation free energy, and conformational entropy upon binding, respectively. More specifically,

$$\Delta E_{MM} = \Delta E_{int} + \Delta E_{ele} + \Delta E_{vdW} \quad (5)$$

$$\Delta G_{sol} = \Delta G_{PB} + \Delta G_{SA} \quad (6)$$

ΔE_{MM} includes the changes in the internal energy ΔE_{int} (due to bonded interactions), electrostatic energies ΔE_{ele}, and the van der Waals energies ΔE_{vdW}. ΔG_{sol} is the sum of the electrostatic solvation energy ΔG_{PB} and the nonpolar term ΔG_{SA} between the solute and the continuum solvent. ΔG_{sol} is calculated using the Poisson-Boltzmann model, while the nonpolar energy is estimated on the basis of the accessible surface area of the proteins.

In our calculations, we neglected the changes in conformational entropy, focusing therefore on the enthalpic contributions to the binding energy. These were estimated with the *g_mmpbsa* tool [2]. A convergence analysis was performed to estimate the appropriate number of frames (Figure S1), which are extracted from the trajectory at regular intervals. The average values and the corresponding errors are obtained with a bootstrap analysis. With ~1500 frames, the result is fully converged with an acceptable uncertainty.

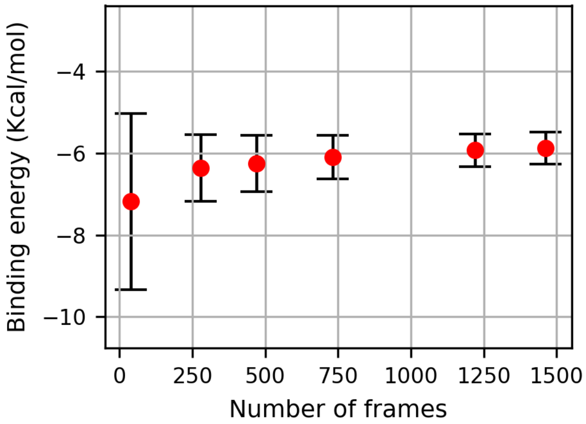

Figure S1: Convergence of the binding energy computed with the MMPBSA method as a function of the number of frames employed for the calculations.

Network analysis As explained in detail in the main text, the protein network is divided in communities, in order to identify functional subdomains of the antibody. As a measure of the quality of the community structure, the modularity parameters Q was

calculated (Table S1)). Q represents the difference in probability of intra- and inter-community connections for a given community repartition, and is defined as:

$$Q = \sum_i \left(e_{ij} - a_i^2\right) \quad (7)$$

where e_{ij} is the fraction of edges that links nodes in community i to nodes in community j, and $a_i = \sum_j e_{ij}$ is the fraction of edges from all communities that connect to nodes belonging to community i. The range of modularity values is between 0 and 1. Values close to 1 identify high-quality community structures, favouring connections intra-community with respect to inter-communities ones.

Table S1: Modularity of the community structures

System	Cluster ID	Modularity
Apo	0	0.8506
	1	0.8486
	2	0.8525
	3	0.8475
	4	0.8474
	5	0.8548
Holo	0	0.8517
	1	0.8557
	2	0.8575
	3	0.8585

All clusters from apo and holo simulations show similar values of modularity, which is very high in all the cases (Table S1). The above-average values (which are usually found between 0.4-0.7 for protein networks) can be explained on the basis of the natural partition of antibodies in structural domains.

Moreover, communities were used as a mean to evaluate the suitability of the cutoffs defining the edges. In order to evaluate the effect of the choice of the cutoffs on the resulting communities, the Community Repartition Difference (CRD) is calculated. The CRD between two network repartitions c_1 and c_2 is defined as:

$$CRD(c_1, c_2) = 1 - \frac{\sum_{n_i,n_j} z(n_i, n_j, c_1) z(n_i, n_j, c_2)}{\sum_{n_i,n_j} z(n_i, n_j, c_1)} \quad (8)$$

where $z(n_i, n_j, c_k)$ is defined as 1 if nodes n_i and n_j belong to the same community in a given network partition c_k, and 0 otherwise. A value of CRD equal to 0 indicates identical repartitions, while a value equal to 1 corresponds to the case of totally different communities. We computed CRD values for several combinations of distance/frame cutoffs, with respect to the reference case of 4.5 Å distance cutoff and 75% frame cutoff (Figure S2). These two values, which are in the range commonly used for protein network analyses [3], are the ones chosen for this study. In all cases, the value of CRD is below 0.2, indicating that a change of the parameters within the range investigated does not lead to substantial changes in the resulting community repartitions.

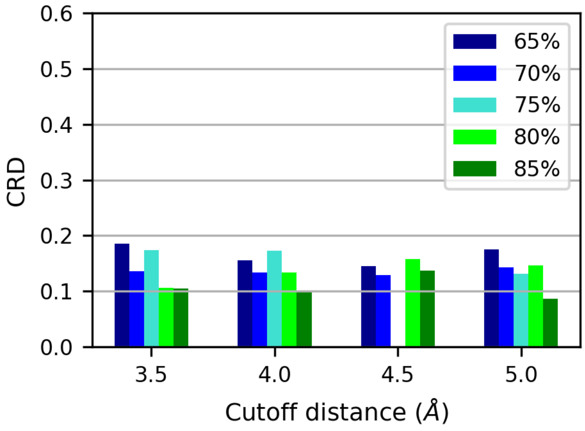

Figure S2: Community Repartition Difference (CRD) computed for different choices of the parameters defining the edges of the network. The "distance cutoff" (ranging here from 3.5 Å to 5.0 Å defines the maximum distance for which two atoms are considered in contact, while the "frame cutoff" (ranging here from 65% to 85%) defines the percentage of frames in which the contact is formed. The values reported in the plot refer to the CRD between each of the possible combination of the two cutoffs and the reference case with 4.5 Å distance cutoff and 75% frame cutoff.

Images Images of the proteins were produced by using VMD [4] and Protein Imager [5], and the graphs were made with python libraries.

S2 Additional figures

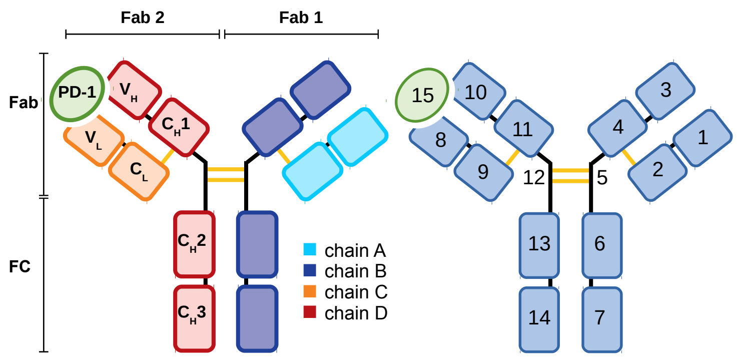

Figure S3: Schematic representation of the antibody pembrolizumab in complex with antigen PD-1, with the indication of the name of each structural domain. Chains C and D correspond to chains F and G in the original PDB file, respectively [6]. The numbering on the right corresponds to the indices used in the correlation matrices.

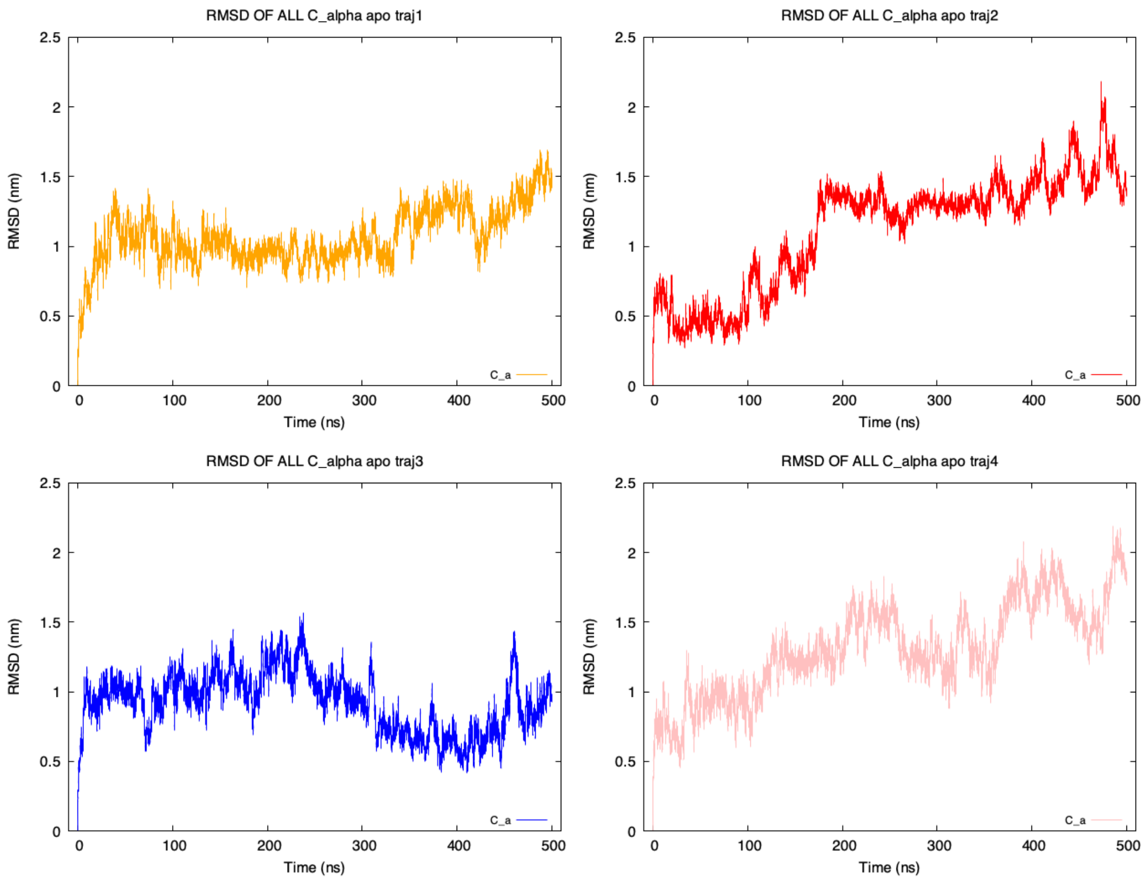

Figure S4: RMSD of the C_α atoms calculated for the full antibody in the apo state, for each of the 500ns-long runs. The large, sudden increase in RMSD in the first few ns of simulation corresponds to the relaxation of the starting structure to a more equilibrated state; from there, the large fluctuations observed correspond to the different conformational changes sampled during the simulations.

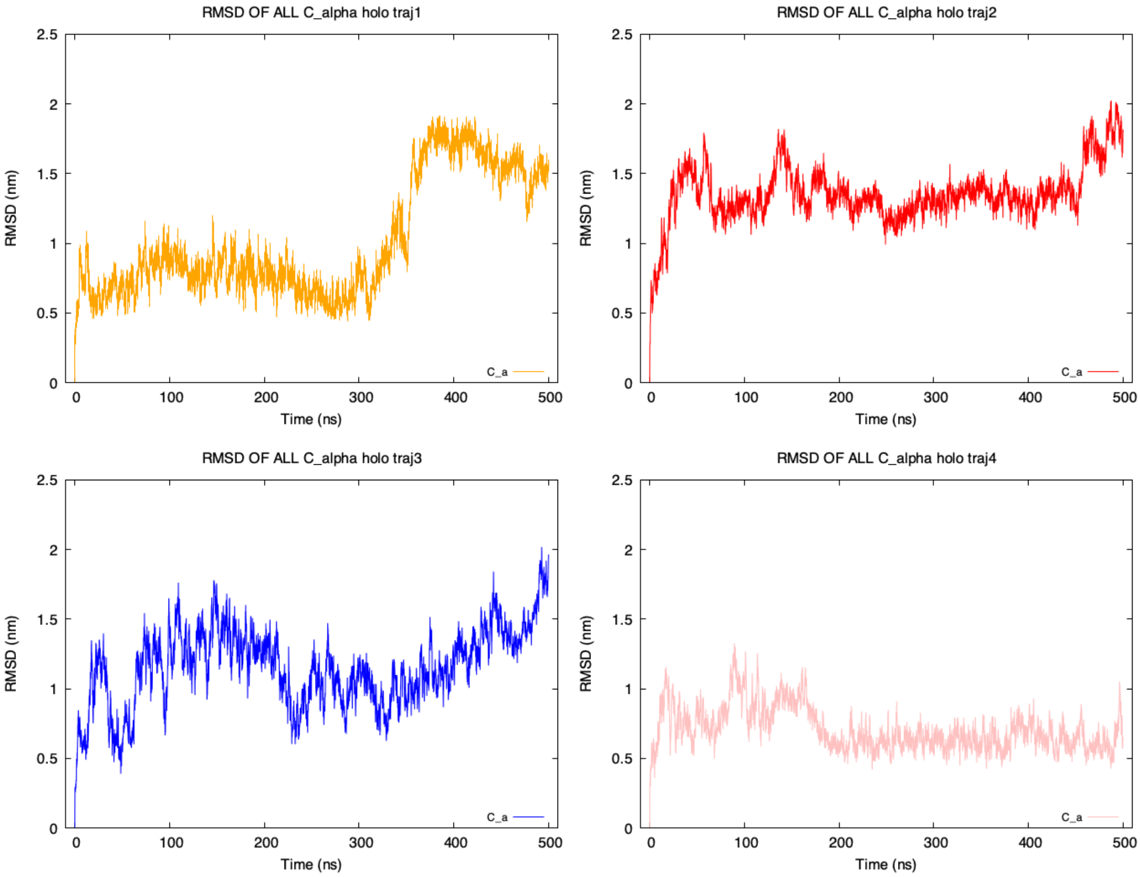

Figure S5: RMSD of the C_α atoms calculated for the full antibody in the holo state, for each of the 500ns-long runs. The large, sudden increase in RMSD in the first few ns of simulation corresponds to the relaxation of the starting structure to a more equilibrated state; from there, the large fluctuations observed correspond to the different conformational changes sampled during the simulations.

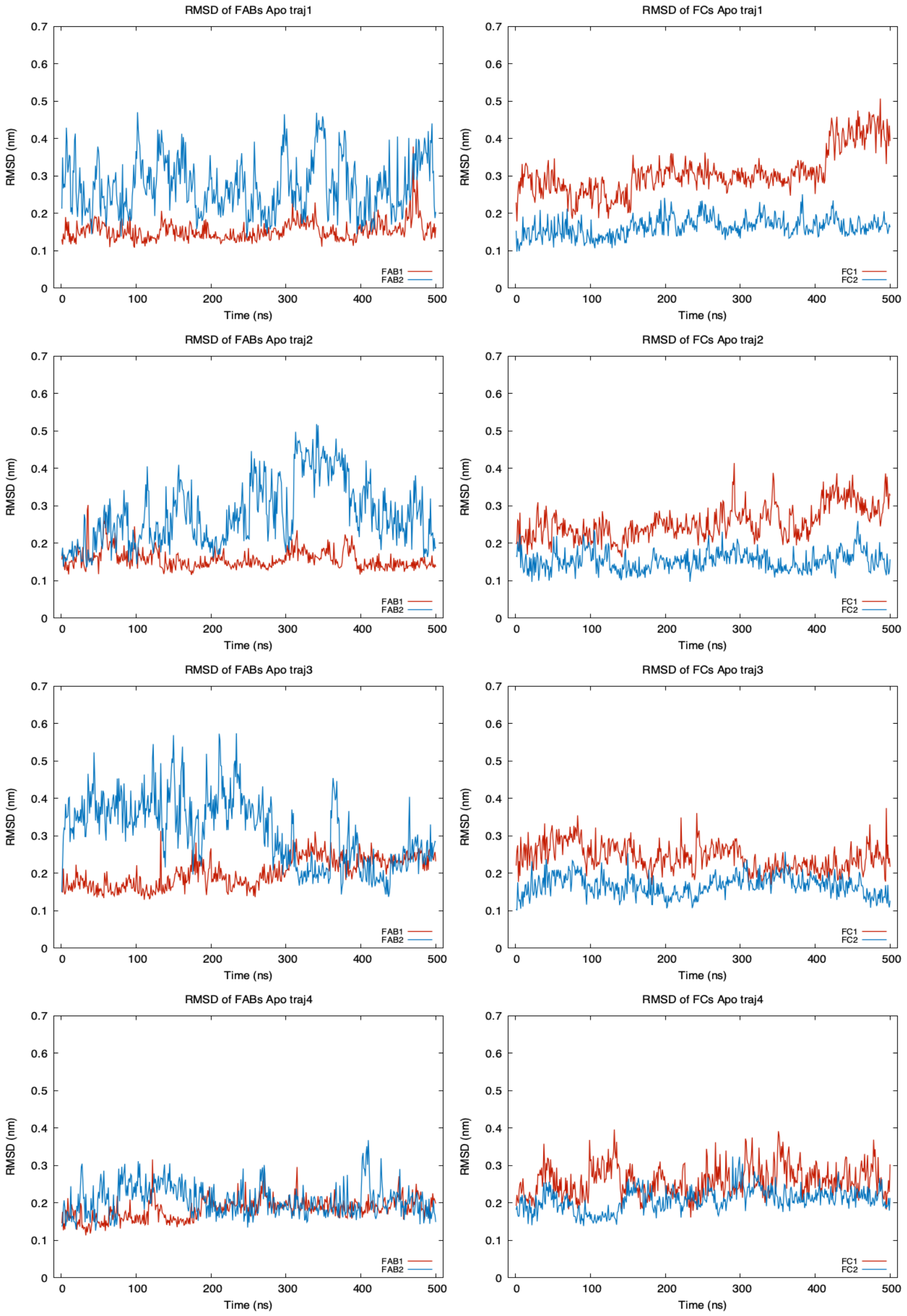

Figure S6: RMSD of the C_α atoms calculated for each structural domain of the antibody, in the four 500ns-long trajectories of the apo state.

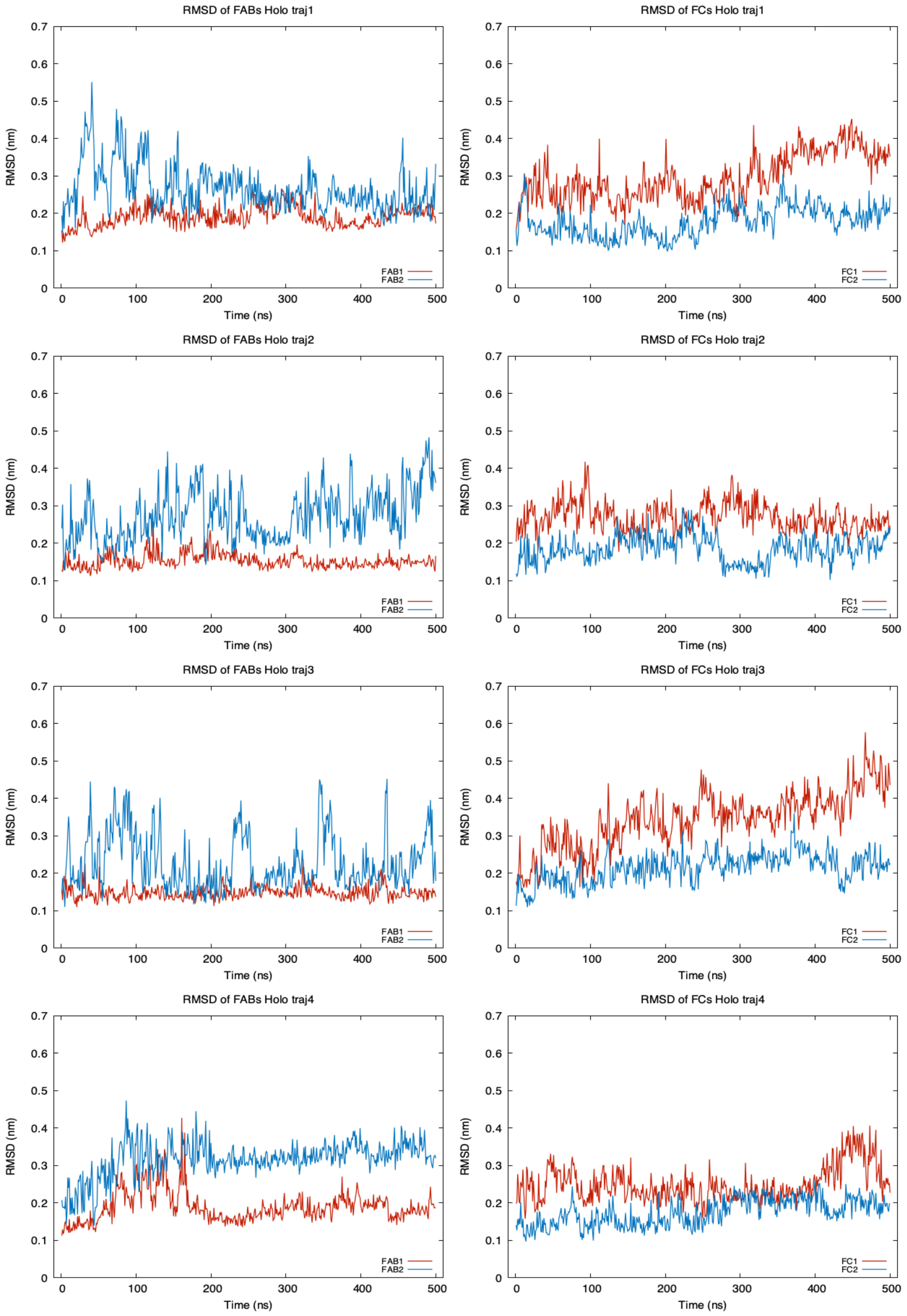

Figure S7: RMSD of the C_α atoms calculated for each structural domain of the antibody, in the four 500ns-long trajectories of the holo state.

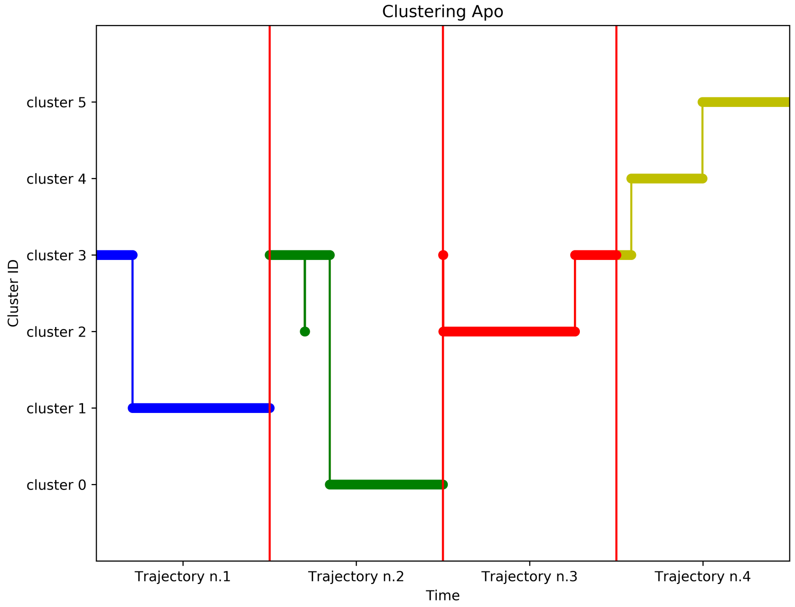

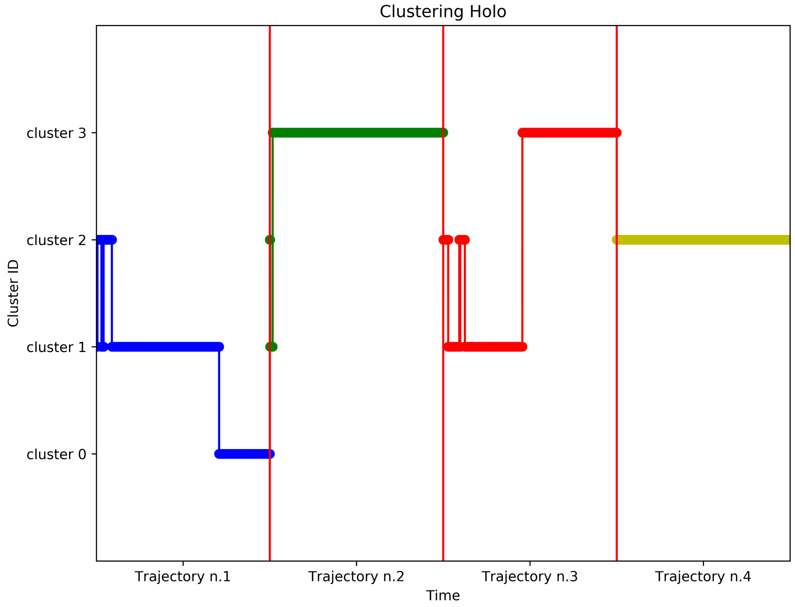

Figure S8: Timeline of the clustering assignment, in the apo (*top*) and holo (*bottom*) systems. Each system is simulated for a total of 2 μs, in four independent replicas of 500 ns; colors are used to distinguish different trajectories.

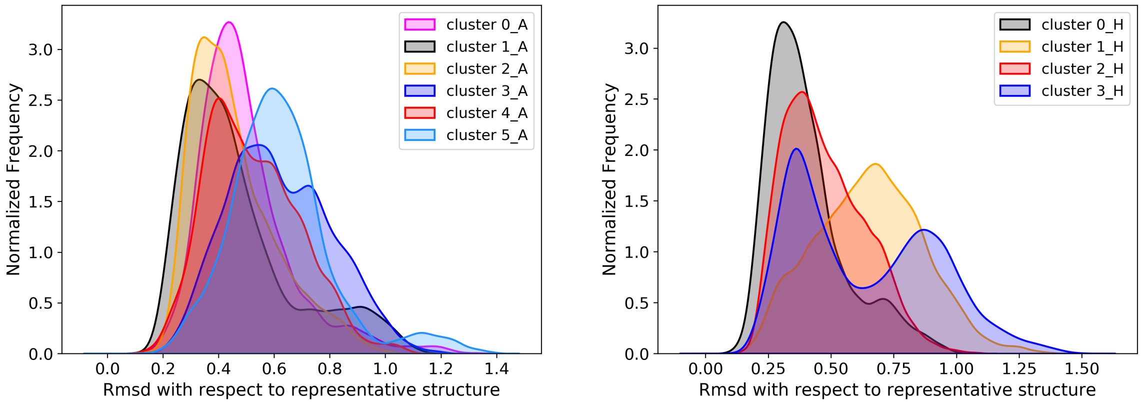

Figure S9: RMSD distribution of antibody structure, for each cluster with respect to the representative conformation. Left: Apo case. Right: Holo case. The threshold used to distinguish among clusters is 1.2 nm. In the UPGMA hierarchical clustering employed, such threshold does not refer to the pairwise distance between observations; instead, it represents the average distance between elements of each cluster [7]. This explains the presence of a few RMSD values larger than 1.2 nm within the same cluster.

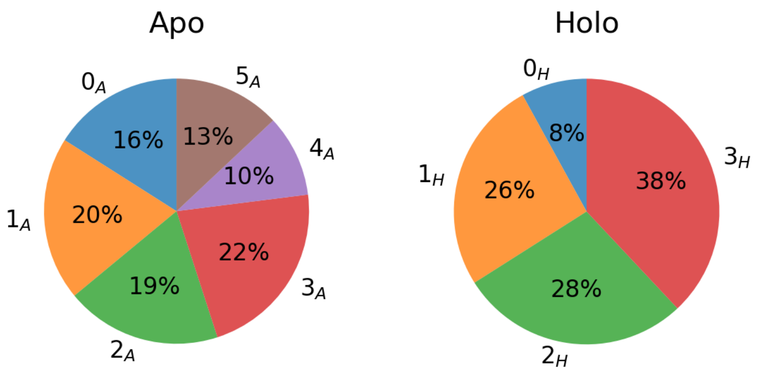

Figure S10: Overall occurrence of the conformational clusters in the apo and holo states.

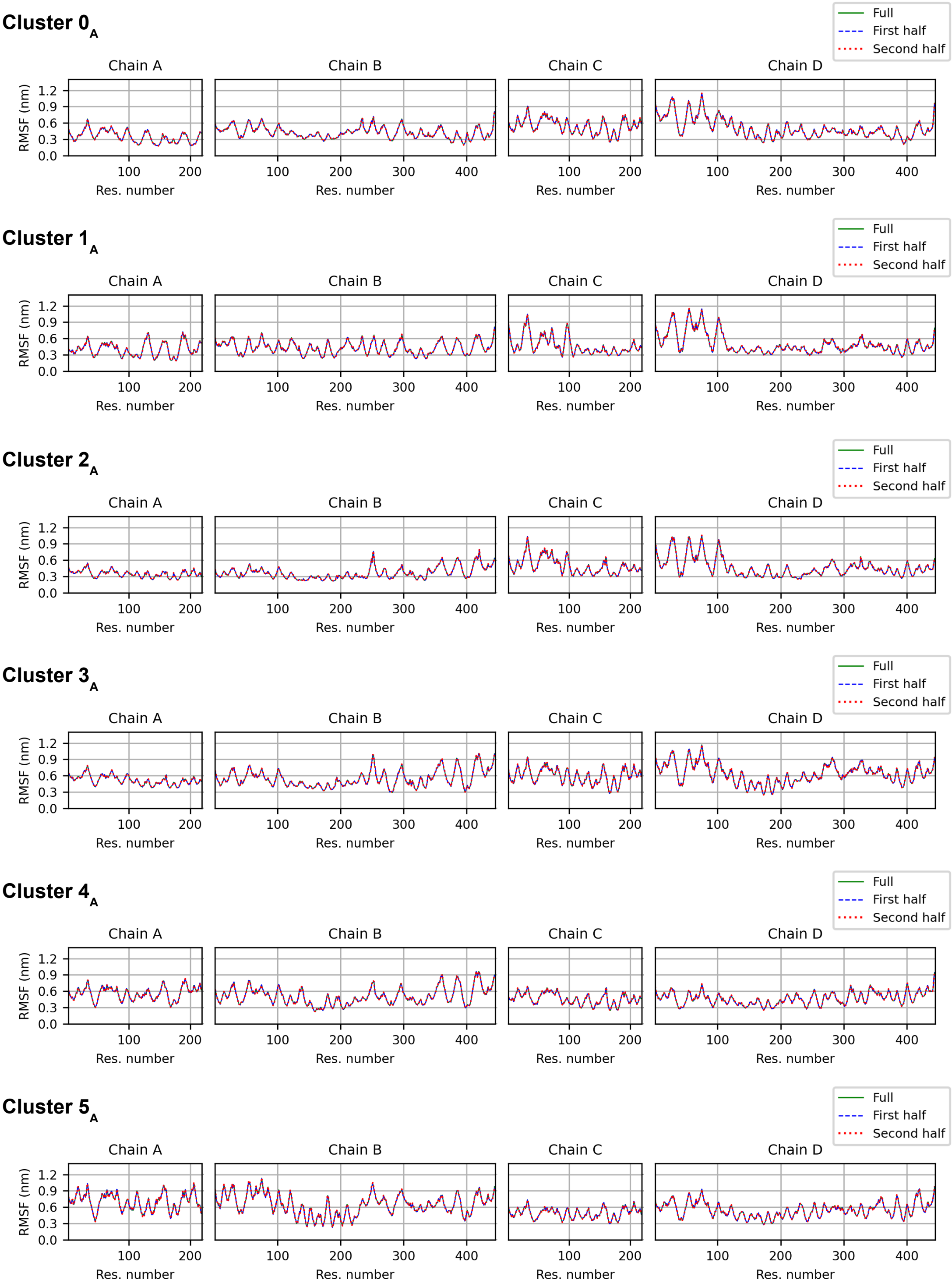

Figure S11: Root-mean-square fluctuations (RMSF) of the C_α atoms, for each cluster of the antibody in the apo state. To assess convergence of the results, frames belonging to the same cluster have been shuffled by randomly changing their order; the RMSF has later been computed on the full, shuffled cluster population and on its two halves. Differences are typically within the line width.

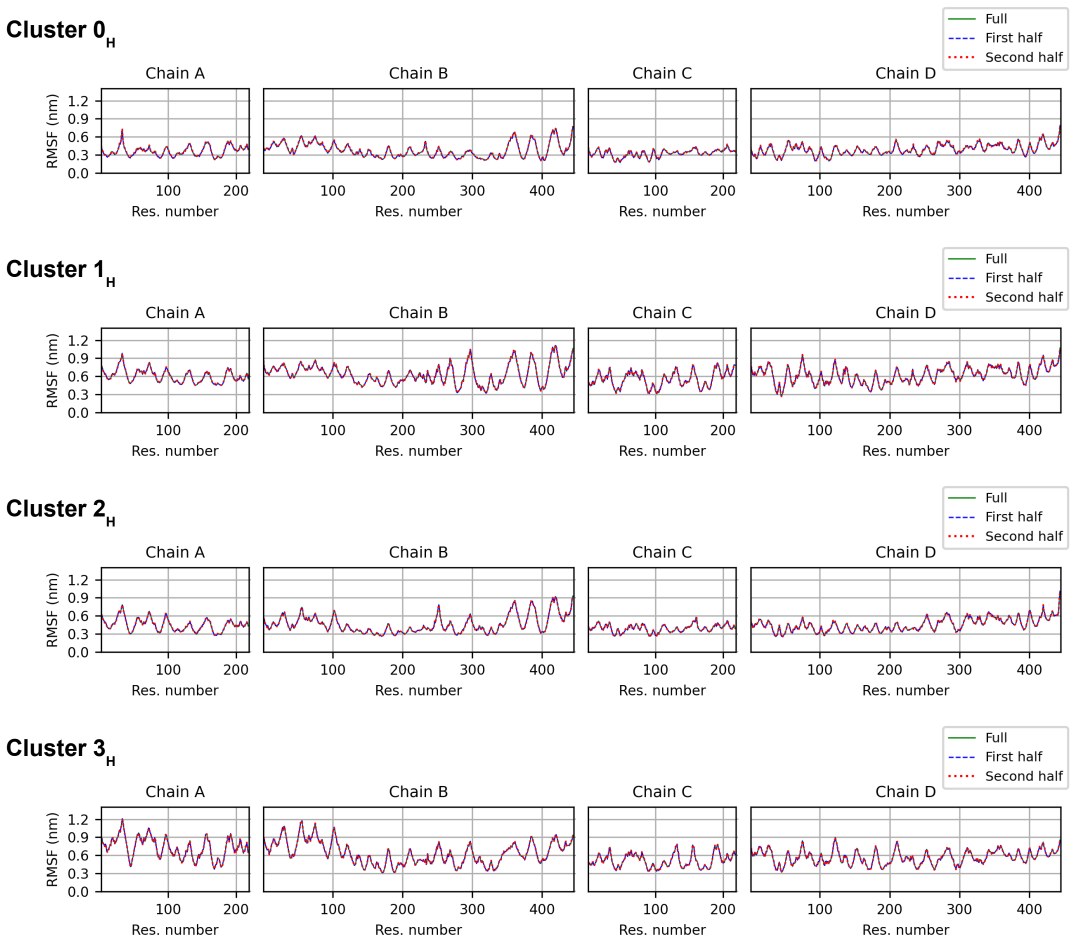

Figure S12: Root-mean-square fluctuations (RMSF) of the C_α atoms, for each cluster of the antibody in the holo state. To assess convergence of the results, frames belonging to the same cluster have been shuffled by randomly changing their order; the RMSF has later been computed on the full, shuffled cluster population and on its two halves. Differences are typically within the line width.

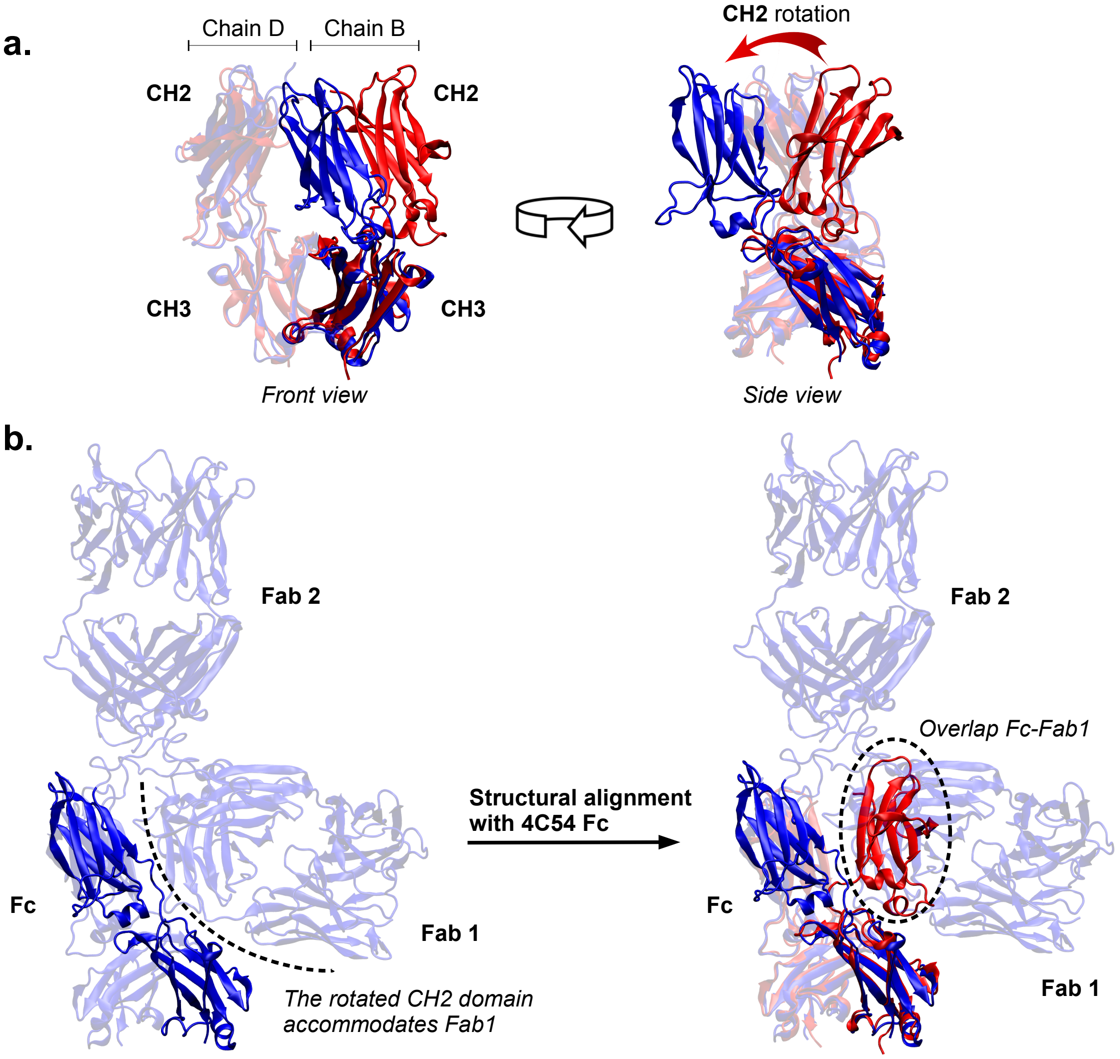

Figure S13: **a.** Comparison of Fc domains from pembrolizumab in the compact conformation (representative structure of cluster 0_A, *blue*) and from an isolated IgG4 Fc (PDB ID: 4C54, *red*), after structural alignment. Chain B is solid color, while chain D is semi-transparent. **b.** Role of the rotated CH2 domain of pembrolizumab in the compact conformations. The close contact between Fab1 and Fc observed in the simulations would not be possible without the peculiar CH2 position; the conformation of CH2 found in 4C54 would lead to an overlap between Fc and Fab1. The images were generated with VMD [4] (`http://www.ks.uiuc.edu/Research/vmd/`).

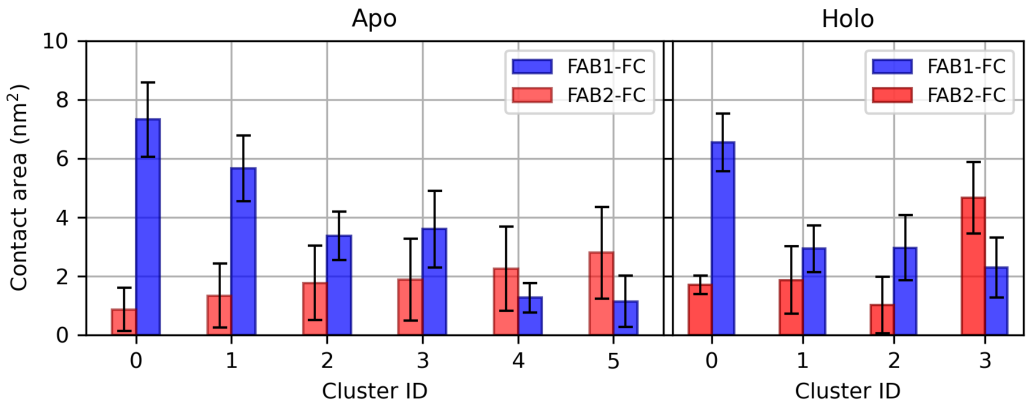

Figure S14: Average contact area between the Fab and the Fc domains, for each conformational cluster in the apo (*left*) and holo (*right*) states.

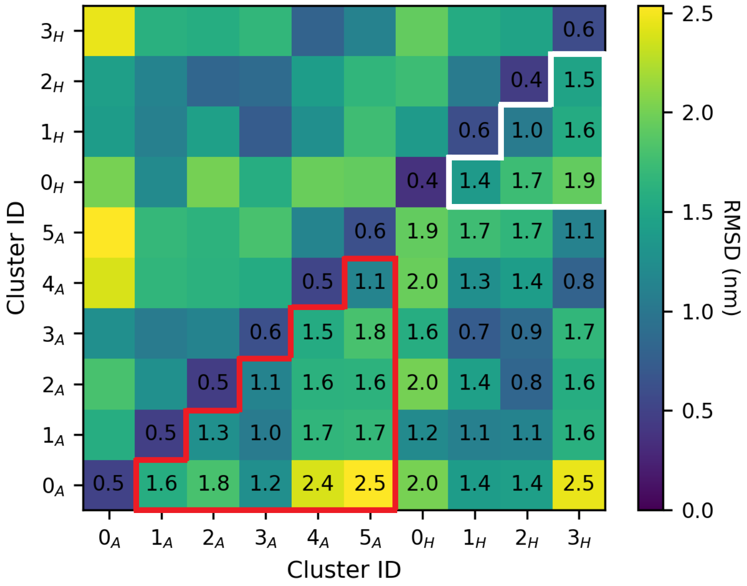

Figure S15: Average RMSD between frames belonging to each pair of clusters. The RMSD has been computed on all the C$^{\alpha}$ atoms of the antibody after structural alignment. Cells within the red border compare clusters of the apo system, while cells within the white border refer to the holo system.

S2.1 Principal components analysis

Cluster 0_A

Fab1 and Fc move apart from Fab2, while Fab1 and Fc remain in close contact during the movement, giving rise to a large contact surface. Fc is rotating in such a way that CH2 of chain B is moving apart from CL of Fab2.

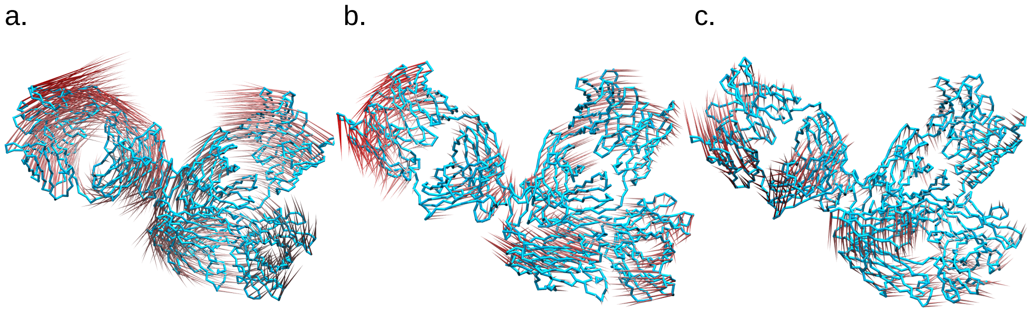

Figure S16: Porcupine representations of modes 1 (**a**), 2 (**b**) and 3 (**c**). The images were generated with VMD [4] (`http://www.ks.uiuc.edu/Research/vmd/`).

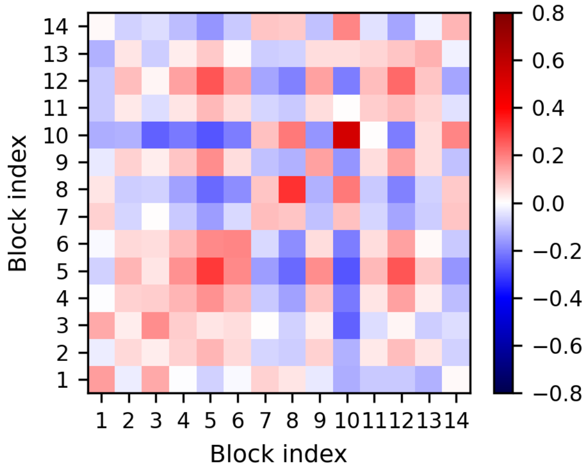

Figure S17: Collinearity of the structural domains in the first three modes.

Cluster 1_A

We observed a high mobility of the variable region of Fab2, in particular VH. The dynamics is similar to the one of cluster 2_A, but this time the CH3 domains tend to move in the same direction of the variable domains of Fab2.

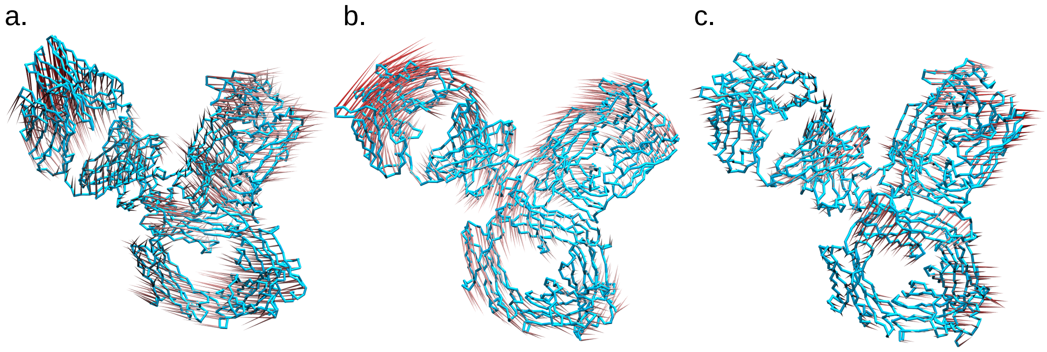

Figure S18: Porcupine representations of modes 1 (**a**), 2 (**b**) and 3 (**c**). The images were generated with VMD [4] (`http://www.ks.uiuc.edu/Research/vmd/`).

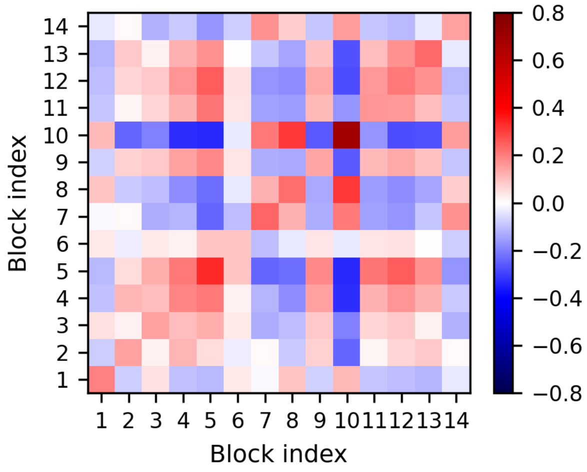

Figure S19: Collinearity of the structural domains in the first three modes.

Cluster 2_A

A deformation of Fab2 takes place: the variable domains of Fab2 move in a strongly anticorrelated motion with respect to the rest of chain D (whose directions of motion are highly parallel), and to the hinge of chain B.

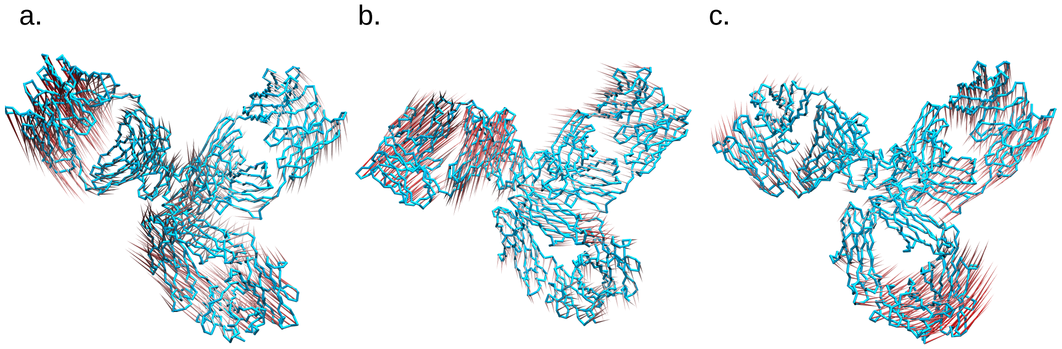

Figure S20: Porcupine representations of modes 1 (**a**), 2 (**b**) and 3 (**c**). The images were generated with VMD [4] (`http://www.ks.uiuc.edu/Research/vmd/`).

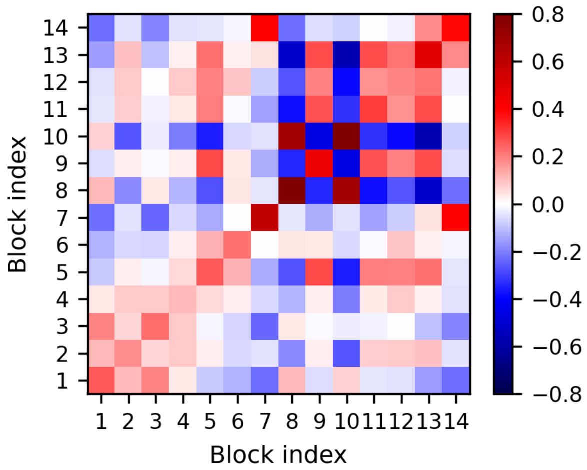

Figure S21: Collinearity of the structural domains in the first three modes.

Cluster 3_A

Rotation of Fab2 takes place until the CL is in contact with the Fc (indeed, this is the only one among the compact clusters in which atomistic non-bonded interactions are formed between residues belonging to these two domains). This happens by means of parallel motions of the CH3 domains and the variable domains in Fab2. At the same time, the distance between the lower hinge segments is reduced, inducing a rotation of Fab1 in the opposite direction of Fab2. This movement of Fab1 is parallel to that of the remaining domains in chain D.

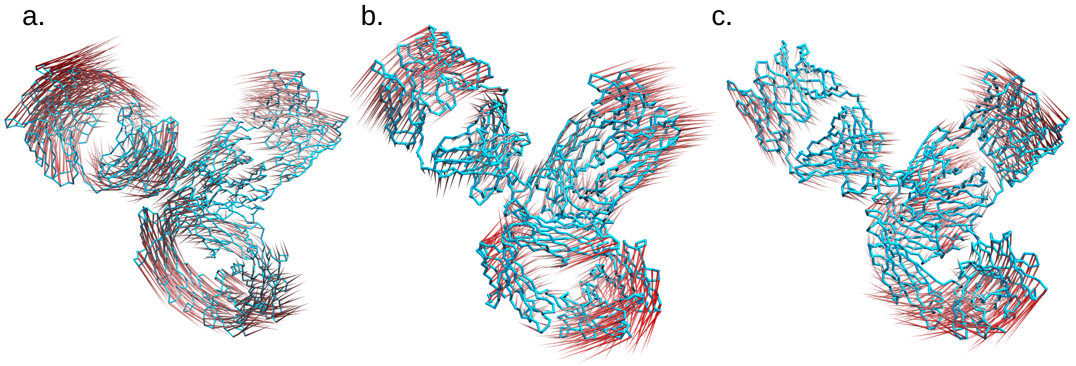

Figure S22: Porcupine representations of modes 1 (**a**), 2 (**b**) and 3 (**c**). The images were generated with VMD [4] (`http://www.ks.uiuc.edu/Research/vmd/`).

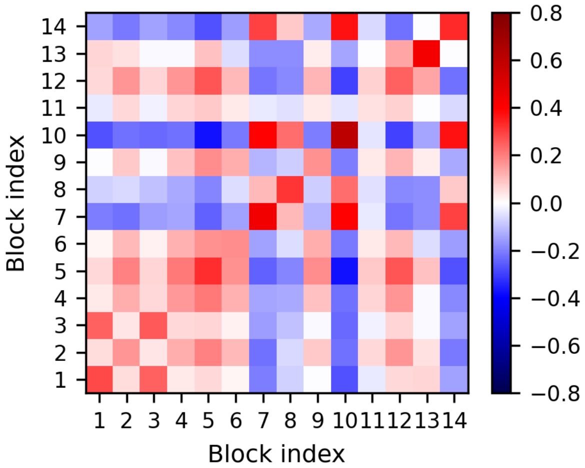

Figure S23: Collinearity of the structural domains in the first three modes.

Cluster 4_A

Fab1 tends to assume a bent conformation, similar to the one in cluster 0_H; the driving force is the concerted motion of CL in chain A, and CH1, hinge and CH2 of chain B. However, the simultaneous rotation of both CH3 domains in Fc prevents the contact of the two domains, and keeps the overall structure open. The rotations of hinge 2 and CH1 in Fab2 are also responsible; they are collinear with the corresponding domains in Fab1, but anticorrelated to the variable region of Fab2.

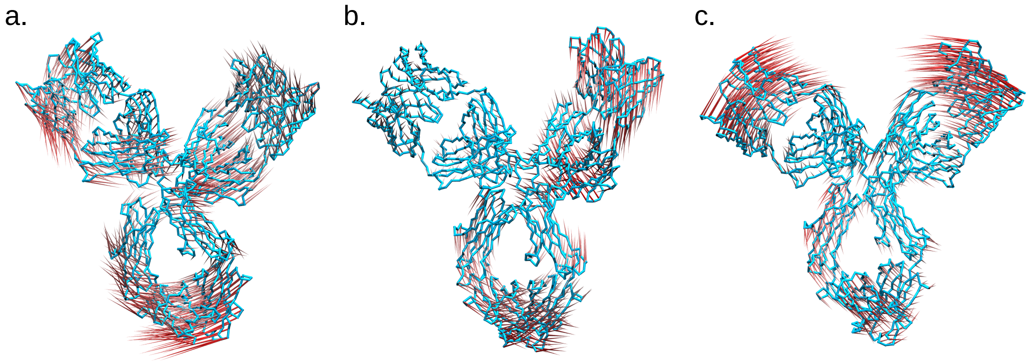

Figure S24: Porcupine representations of modes 1 (**a**), 2 (**b**) and 3 (**c**). The images were generated with VMD [4] (`http://www.ks.uiuc.edu/Research/vmd/`).

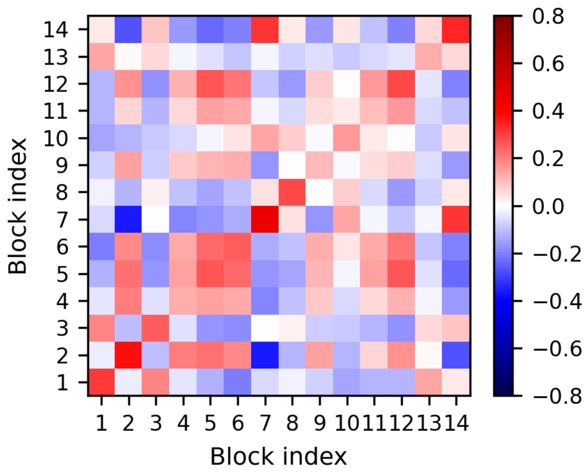

Figure S25: Collinearity of the structural domains in the first three modes.

Cluster 5_A

Fab1 rotates up to the point of laying perpendicular with respect to the main axis of Fc, giving to the immunoglobulin an overall T shape. This is facilitated by the simultaneous antiparallel motion of Fab2 with respect to hinge and CH2 of chain A.

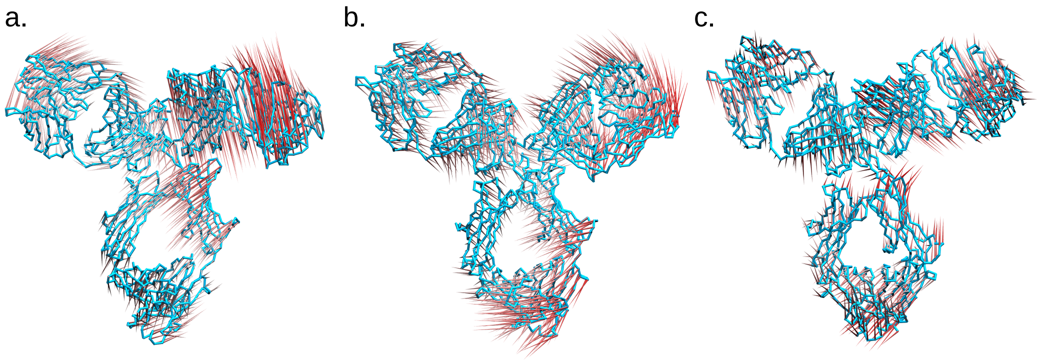

Figure S26: Porcupine representations of modes 1 (**a**), 2 (**b**) and 3 (**c**). The images were generated with VMD [4] (`http://www.ks.uiuc.edu/Research/vmd/`).

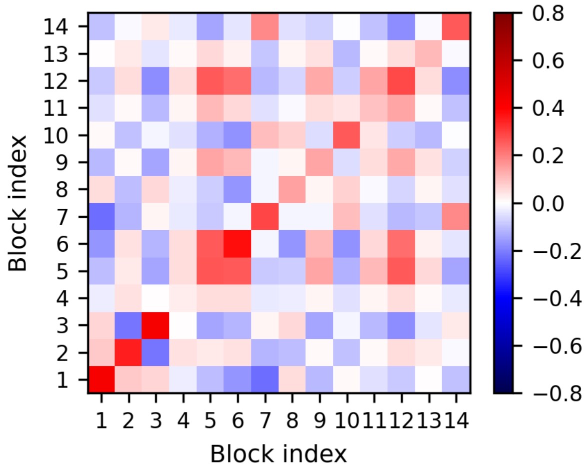

Figure S27: Collinearity of the structural domains in the first three modes.

Cluster 0_H

Fab1 moves in same direction of hinge, and opposite to variable domains of Fab2, in a scissor-like movement (bending). As in other clusters, a correlation between the variable region of Fab2 and the CH3 domains is observed. These movements are allowed by torsion of the lower hinge 2, and of the loop between VH and CH1 of Fab2. The motion giving rise to the peculiar conformation of Fab1 is described by mode 3; it originates from the torsion in the upper hinge of chain 1 (residues LYS^{439}-TYR^{440}-GLY^{441}). The conformation with bent Fab1 is then stabilized by side-chain/side-chain interactions between Fab1 and a turn in CH2 domain.

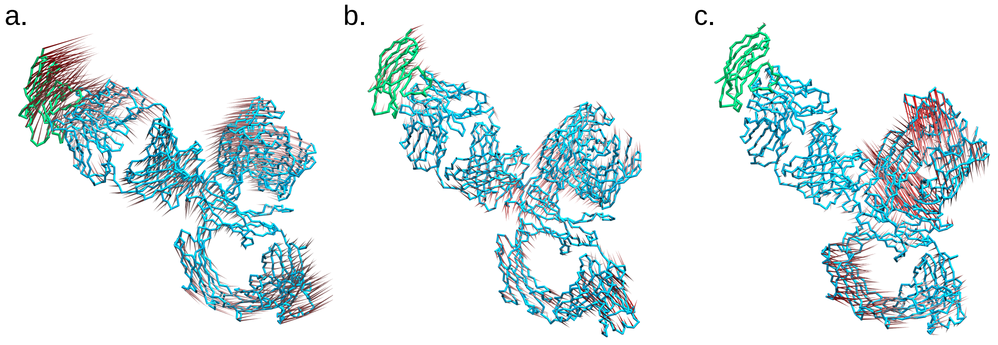

Figure S28: Porcupine representations of modes 1 (**a**), 2 (**b**) and 3 (**c**). In green is the antigen, PD-1. The images were generated with VMD [4] (`http://www.ks.uiuc.edu/Research/vmd/`).

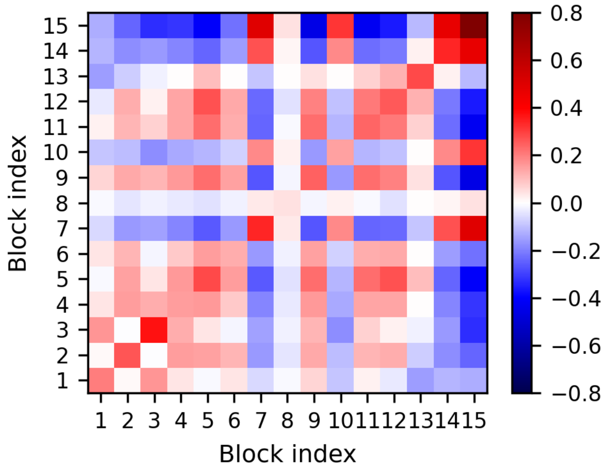

Figure S29: Collinearity of the structural domains in the first three modes.

Cluster 1_H

A strongly parallel movement of the whole chain B is observed (with the usual exception of the CH3 domain). Scissoring of Fab2/Fc, until Fab2 is in contact with CH2 of chain B (many non-bonded interactions are detected, see below). In mode 2, wagging of the two Fabs and the Fc. In mode 3, a twist of the two Fabs along their major axis is observed.

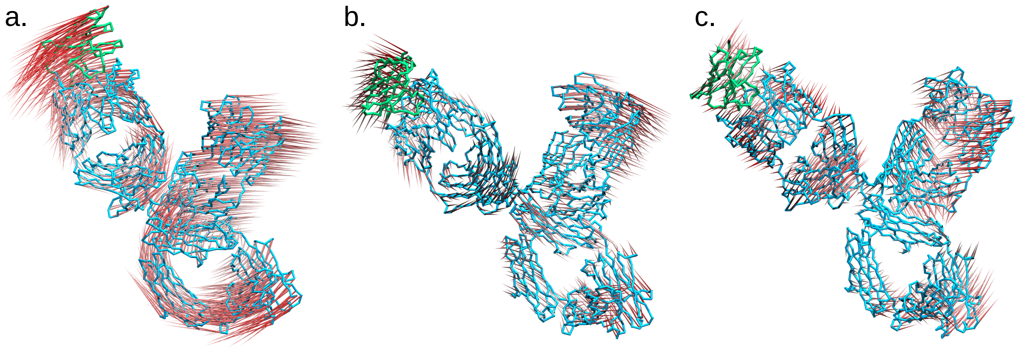

Figure S30: Porcupine representations of modes 1 (**a**), 2 (**b**) and 3 (**c**). In green is the antigen, PD-1. The images were generated with VMD [4] (`http://www.ks.uiuc.edu/Research/vmd/`).

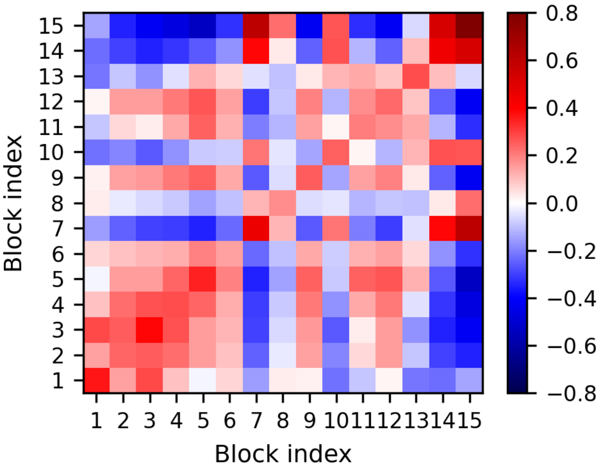

Figure S31: Collinearity of the structural domains in the first three modes.

Cluster 2_H

Similar to the case of cluster 3_H, but with a little role of Fab2 in determining the overall shape of the molecule. Wagging of Fab1 and Fc, with CL and CH2 getting close and far apart, oscillating between open and compact conformations. In mode 2 and 3, instead, Fab1 and Fc maintain a significant contact area, while Fab2 is twisted (the hinge of the motion is located on the loops connecting the variable to the constant domains).

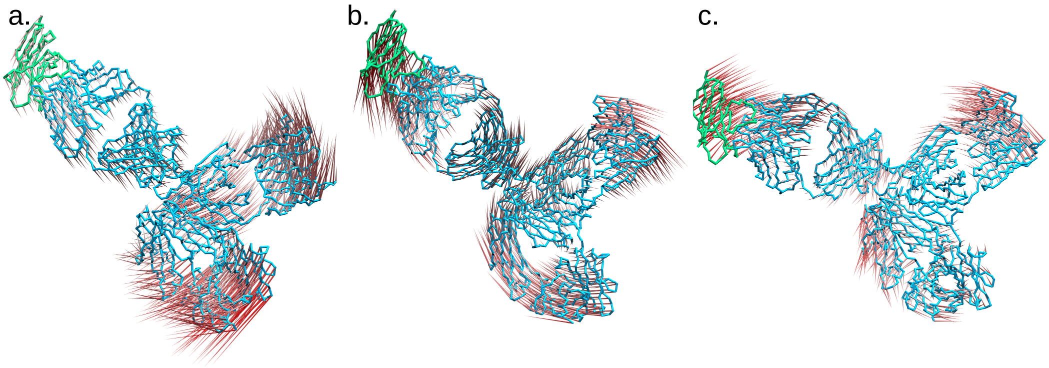

Figure S32: Porcupine representations of modes 1 (**a**), 2 (**b**) and 3 (**c**). In green is the antigen, PD-1. The images were generated with VMD [4] (`http://www.ks.uiuc.edu/Research/vmd/`).

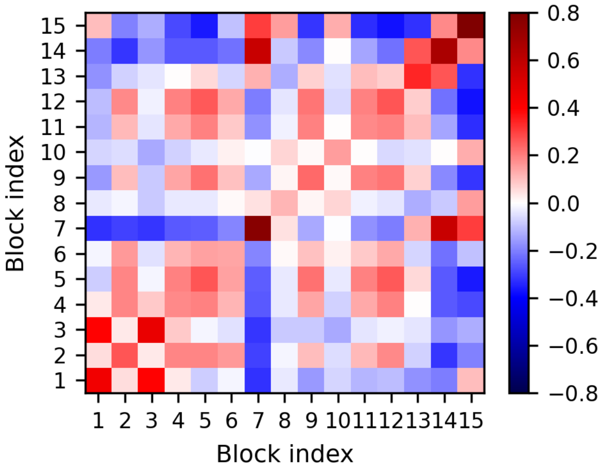

Figure S33: Collinearity of the structural domains in the first three modes.

Cluster 3_H

A bending movement of the two Fabs is observed, accompanied by a bending of the two CH2 domains. An antiparallel movement of VH and hinge/VL of chain B is observed. A slight twist of Fab1 takes place in a manner similar to cluster 0_H.

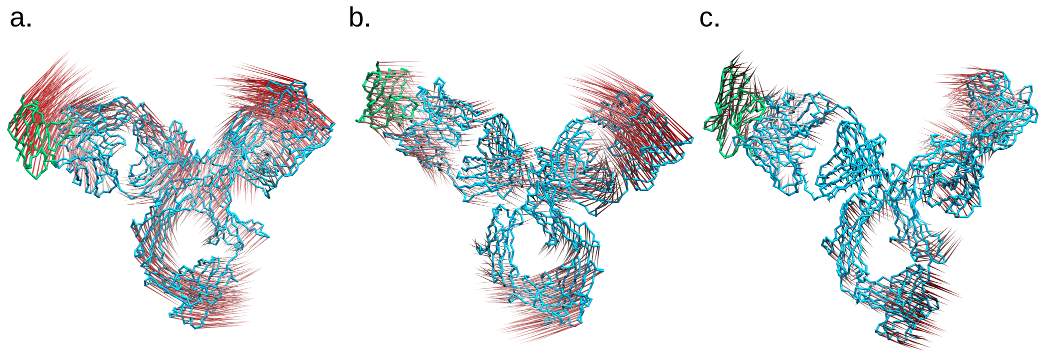

Figure S34: Porcupine representations of modes 1 (**a**), 2 (**b**) and 3 (**c**). In green is the antigen, PD-1. The images were generated with VMD [4] (`http://www.ks.uiuc.edu/Research/vmd/`).

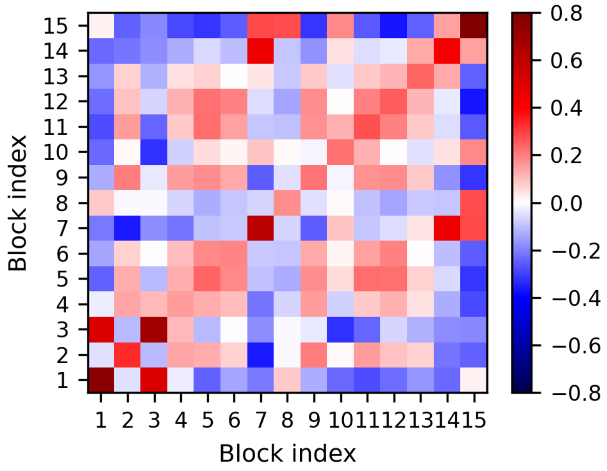

Figure S35: Collinearity of the structural domains in the first three modes.

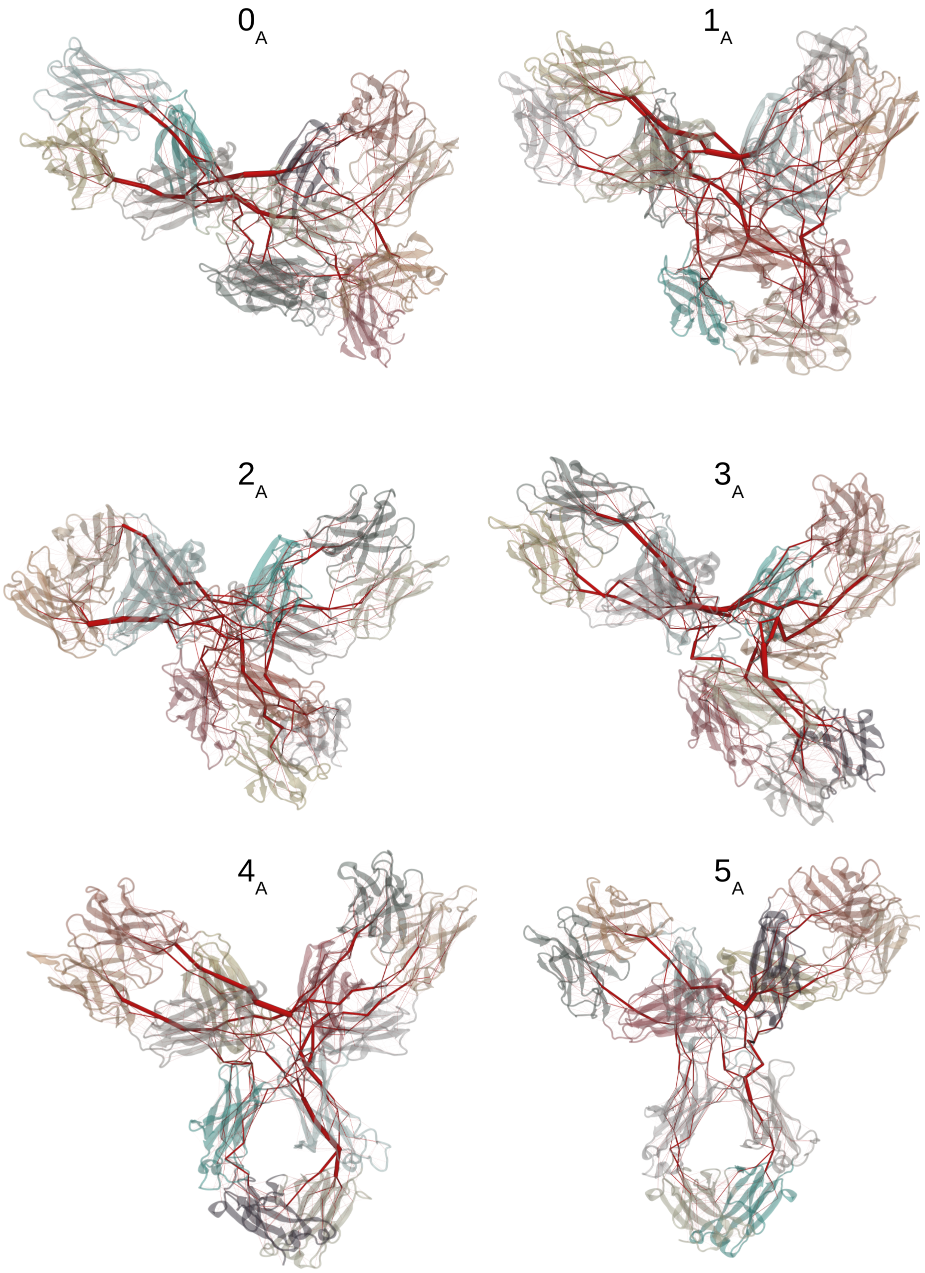

Figure S36: Edges of the apo networks are represented as red lines, with thickness proportional to the edge betweenness. The numbers indicate the cluster ID. The images were generated with VMD [4] (`http://www.ks.uiuc.edu/Research/vmd/`), using the script by Melo et al. for the visualization of the networks [3].

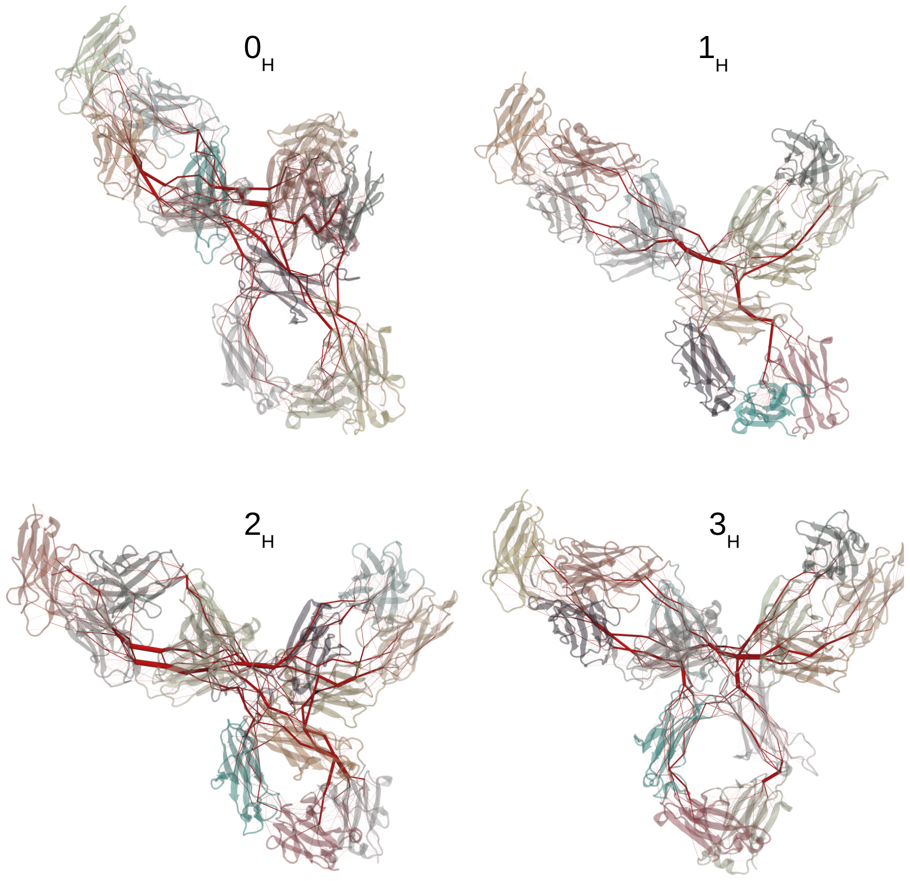

Figure S37: Edges of the holo network are represented as red lines, with thickness proportional to the edge betweenness. The numbers indicate the cluster ID. The images were generated with VMD [4] (`http://www.ks.uiuc.edu/Research/vmd/`), using the script by Melo et al. for the visualization of the networks [3].

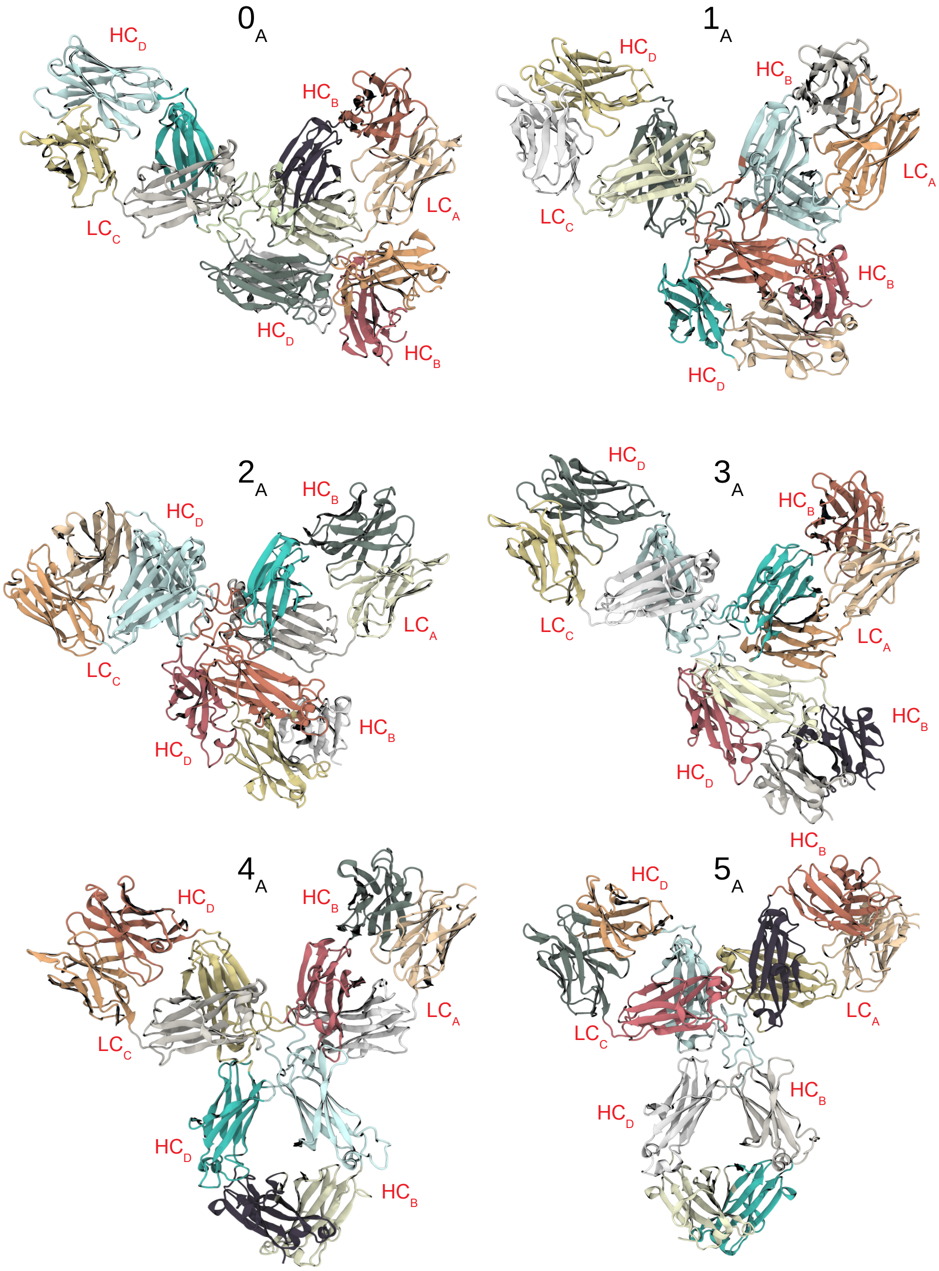

Figure S38: Community network repartitions for the apo system. The numbers indicate the cluster ID, and different colors are used to differenciate the communities within each structure. Heavy and light chains (HC and LC, respectively) are labelled for the Fc and for each Fab. The images were generated with VMD [4] (`http://www.ks.uiuc.edu/Research/vmd/`), using the script by Melo et al. for the visualization of the communities [3].

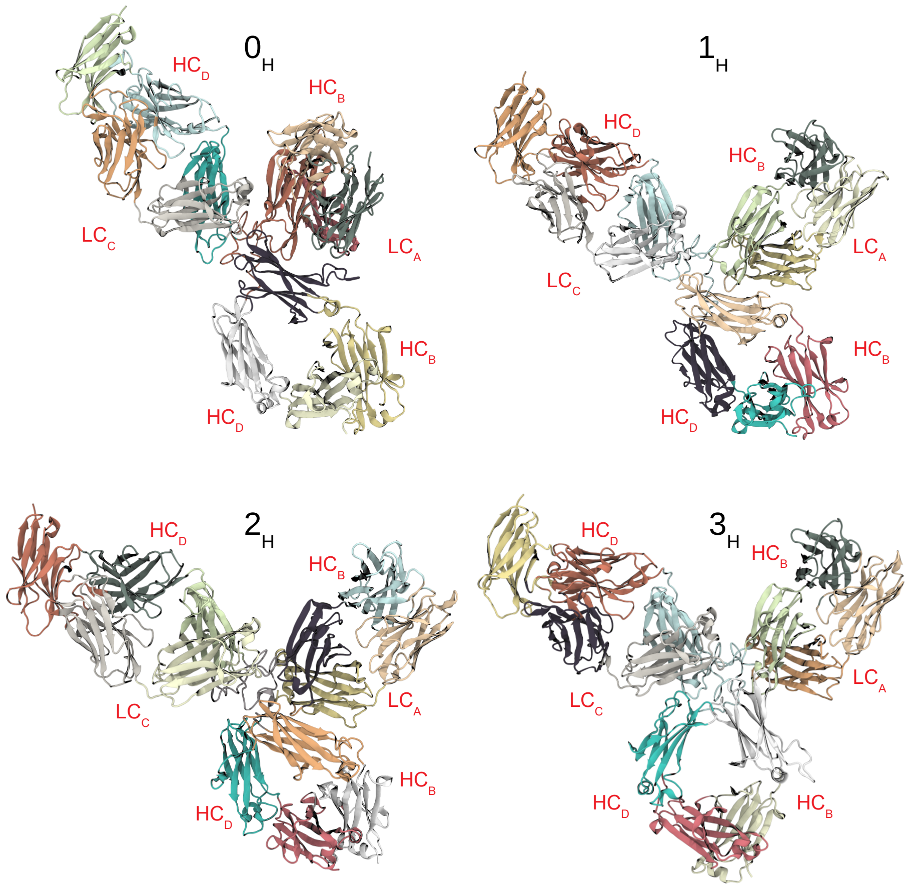

Figure S39: Community network repartitions for the holo system. The numbers indicate the cluster ID, and different colors are used to differenciate the communities within each structure. Heavy and light chains (HC and LC, respectively) are labelled for the Fc and for each Fab. The images were generated with VMD [4] (`http://www.ks.uiuc.edu/Research/vmd/`), using the script by Melo et al. for the visualization of the communities [3].

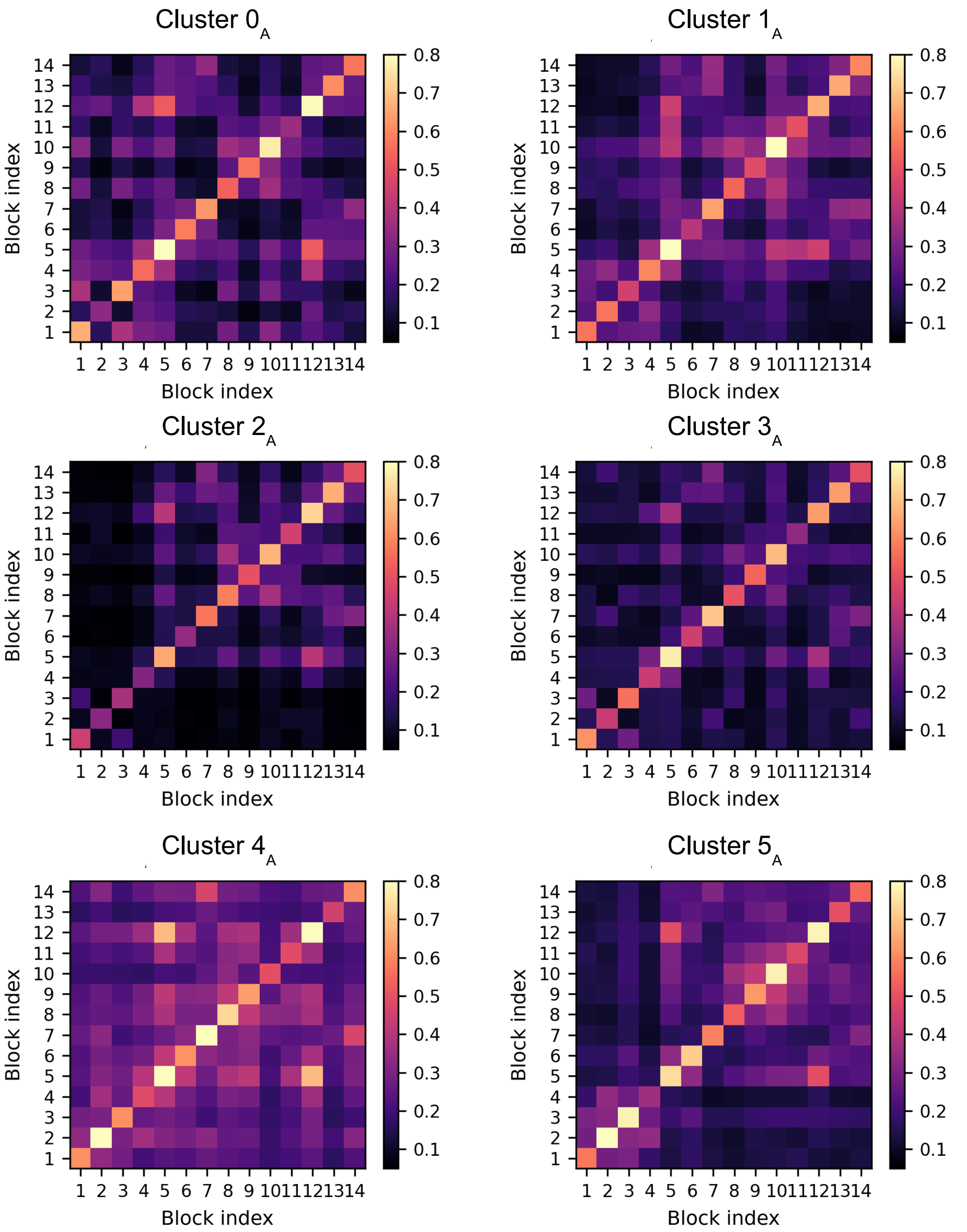

Figure S40: Mutual information averaged within each structural domain of pembrolizumab in the apo state, for each conformational cluster.

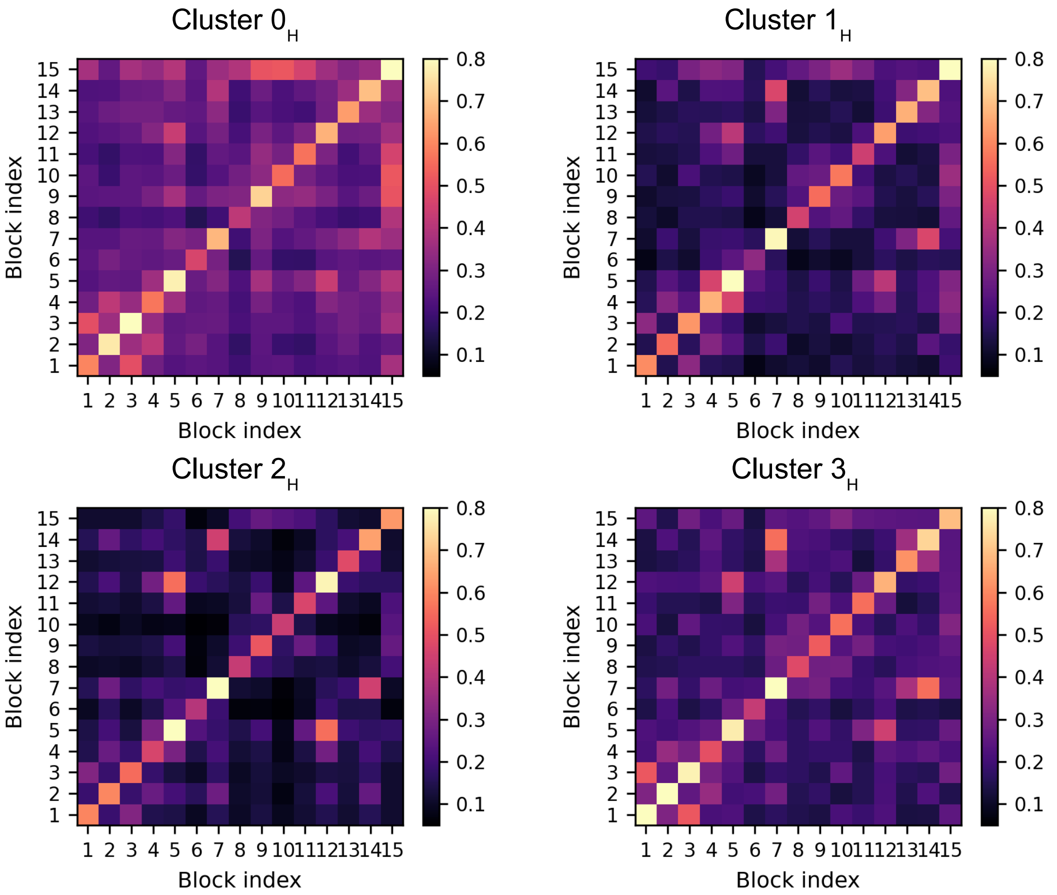

Figure S41: Mutual information averaged within each structural domain of pembrolizumab in the holo state, for each conformational cluster.

S2.2 The hinge region

Hinge 1 (H1), namely the hinge segment of chain B, shows higher correlations than hinge 2 (H2, from chain D) with the rest of the protein, in all the apo and holo clusters. These correlations are particularly strong with the domains of Fab2. Network analysis shows that, in the apo case, a particularly favoured edge is the one between ASP^{126} of CL and LYS^{221} of H1; the former is part of Fab2, and the latter is connecting Fab1 and the hinge segment. Atomistic-level inspection shows the presence of a persistent salt bridge between the two residues in clusters 3_A, 4_A and 5_A.

Edge betweenness highlights the importance of hinge proline residues for the compact apo conformations (cluster 0_A). In particular, PRO^{225} in H1 and PRO^{228} in H2 are key residues for inter-Fabs communication. The former is part of the highly central path ARG^{217}(CH1, Fab1)-PRO^{225}(H1)-GLU^{219}(CH1, Fab2), while the latter (which corresponds to the residue mutated from serine, in order to avoid Fab-arm exchange [8]) is part of CYS^{134}(CH1, Fab1)-PRO^{228}(H2)-TYR^{222}(H2)-LEU^{196}(CH1, Fab2). This last path is observed also in other compact conformations, those of cluster 2_A; however, in this case, the network involving hinge residues is fragmented in a large number of paths with low centrality.

Prolines play a central role also in cluster 1_A, where PRO^{224} in hinge 1 is highly correlated to PRO^{130} on CH1 of Fab2. In addition, PRO^{224} is part of a highly central path connecting hinge 1 with Fab1 through TYR^{222} and THR^{198} of CH1.

The same PRO^{224} of H1 residue is involved in a highly central path in cluster 3_A, where it connects LYS^{221} in CH1 of Fab1 to ALA^{132} in CH1 of Fab2. In this cluster, the path involving residues SER^{220}(H1)-LYS^{444}(H1)-GLU^{2019}(CH1, Fab2) is also highly relevant.

In the holo case we observe that, also in the most open conformations (cluster 3_H), the hinge region still plays an important role for information transfer, in particular when compared to the apo case. Again, prolines appear as key residues. In cluster 0_H, there are no significant interactions between the hinge residues and the nearby domains. In all clusters, none of the residues of H2 is involved in high-centrality contacts. Several paths with highest betweenness include instead H1 residues. In cluster 2_H, pathways with high betweenness include: PRO^{1013}(CH1, Fab2)-SER^{1015}(CH1, Fab2)-PHE^{234}(H1)-GLY^{237}(CH2, chain B), CYS^{218}(CL, Fab2)-LYS^{221}(H1) and PRO^{227}(H1)-CYS^{226}(H2). In clusters 1_H and 3_H there is one path with high centrality crossing the hinge, namely LEU^{196}(CH1, Fab1)-TYR^{222}(H1)-PRO^{227}(H1)-GLU^{219}(CH1, Fab2).

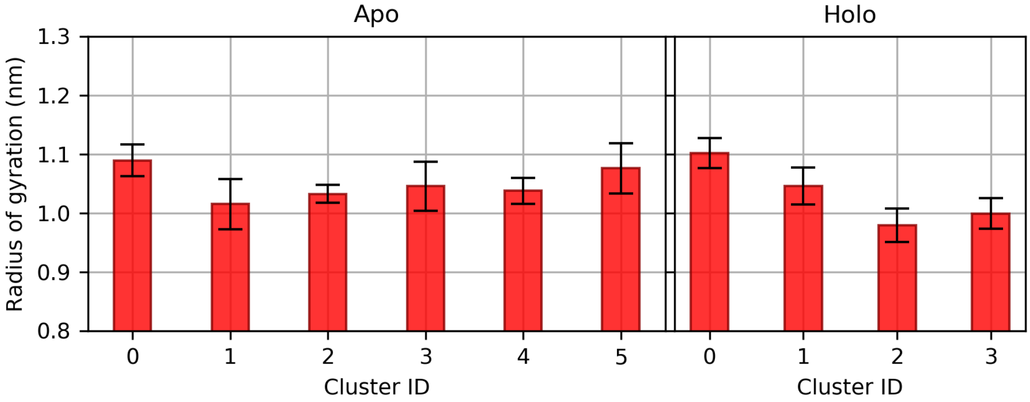

Figure S42: Average radii of gyration of the hinge region, in the apo and holo clusters.

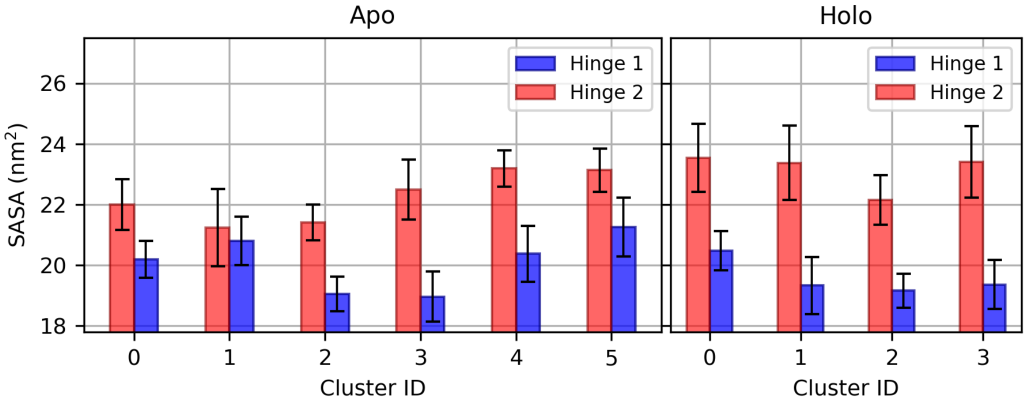

Figure S43: Average solvent accessible surface area (SASA) of hinge segments in chain B (hinge 1) and chain D (hinge 2), in the apo and holo clusters. The values refer to the hinge surface alone, computed without taking into account hindrance from nearby domains. The two segments appear highly asymmetrical; in particular, the smaller SASA for hinge 1 is indicative of a more compact, less accessible conformation.

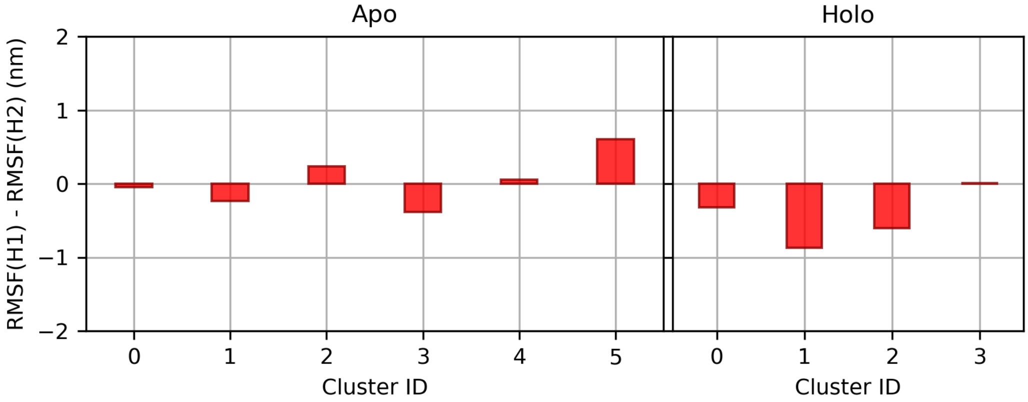

Figure S44: Difference between the total C^{α} RMSF of the two hinge chains, in the clusters of apo and holo states. In the holo case, hinge 1 appears overall less flexible than hinge 2, while in the apo systems this is true only for 2 of the 5 conformational clusters.

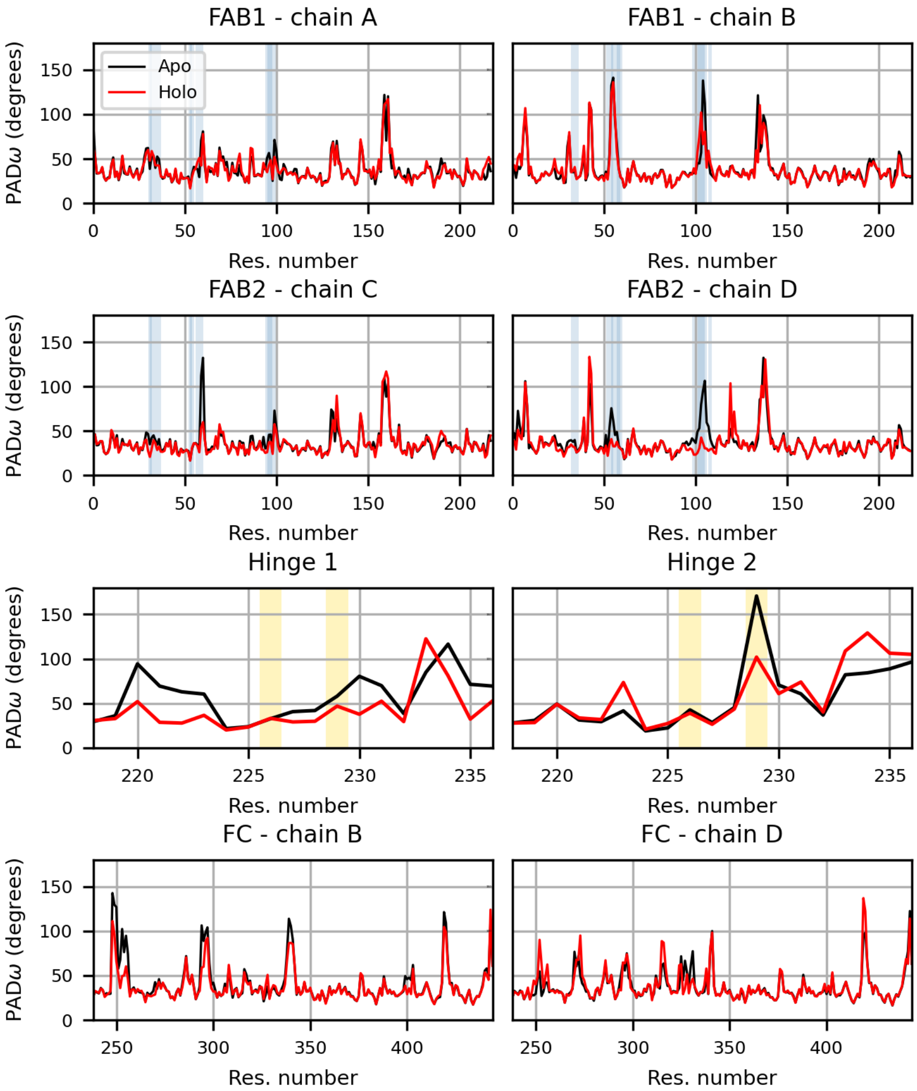

Figure S45: Comparison between the PAD_ω parameter in the apo and holo antibody. PAD_ω measures of backbone torsional plasticity through the spreading of ϕ and ψ dihedral angles. The blue-shaded areas correspond to the residues involved in the binding with PD1; as expected, the bound antigen leads to a decreased flexibility in the paratope of Fab2. The yellow-shaded areas correspond to the cysteine residues forming inter-chain disulfide bonds in the hinge.

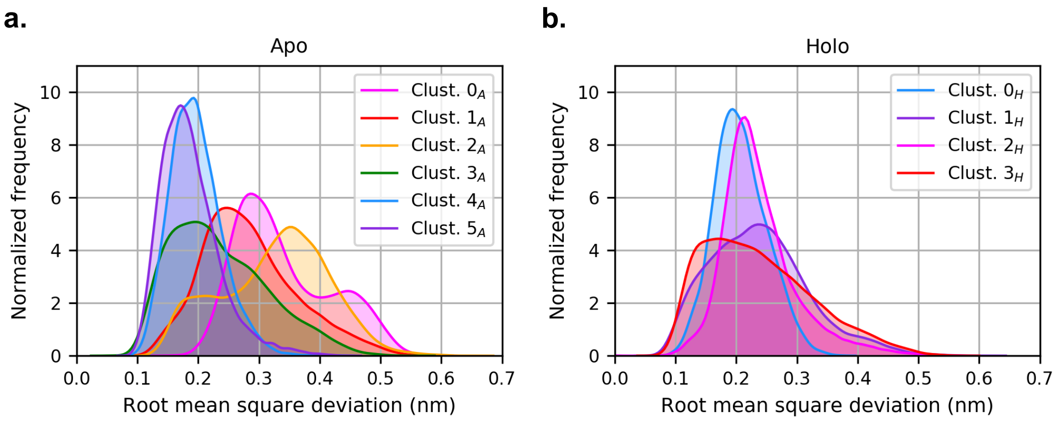

Figure S46: Plots of the RMSD of binding site residues (C^α) in the conformational clusters of the apo (**a**) and holo (**b**) systems, with respect to the crystallographic structure of the Fab/PD-1 complex. The deviations are calculated after fitting the trajectories on the variable region (Fab2) of the antibody. In the holo case, clusters present a more uniform behaviour with respect to the apo system.

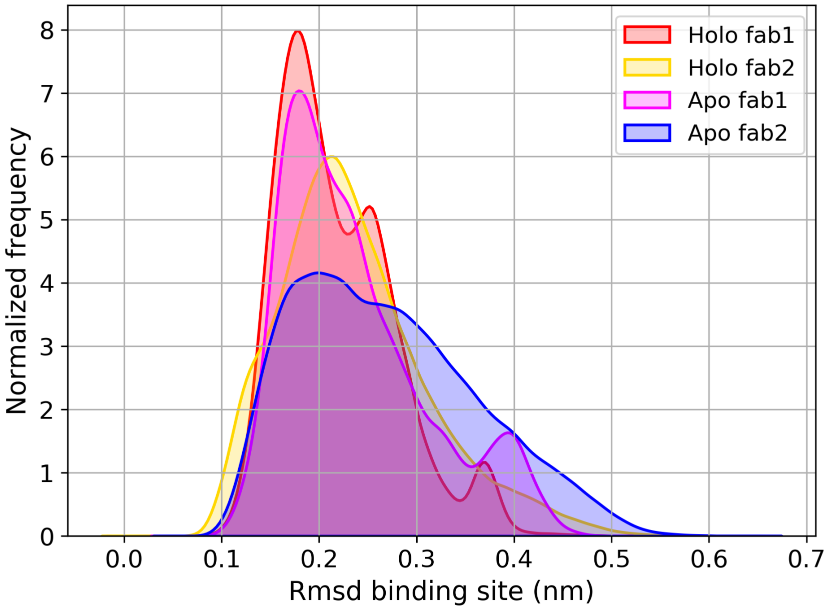

Figure S47: Plots of the RMSD of binding site residues (C_α) on Fab1 and Fab2, in both apo and holo states. In the holo case, only Fab2 is bound to the antigen. The RMSD is computed with respect to the crystallographic structure of the Fab/PD-1 complex.

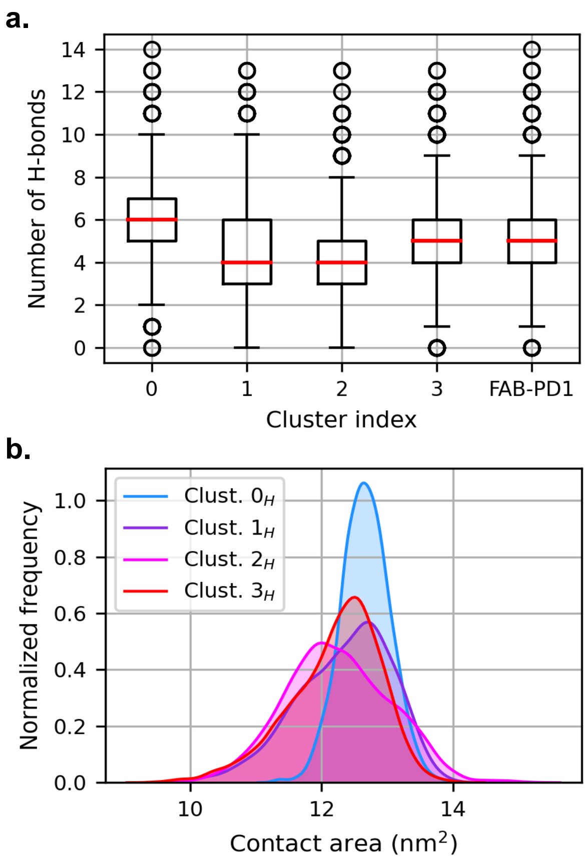

Figure S48: **a.** Comparison between the number of hydrogen bonds between the CDR and the PD1 in the four clusters of the bound antibody, and in the simulation of the antigen with the Fab alone. **b.** Comparison of the contact area distributions between the Fv and the PD1 in the four clusters of the bound antibody.

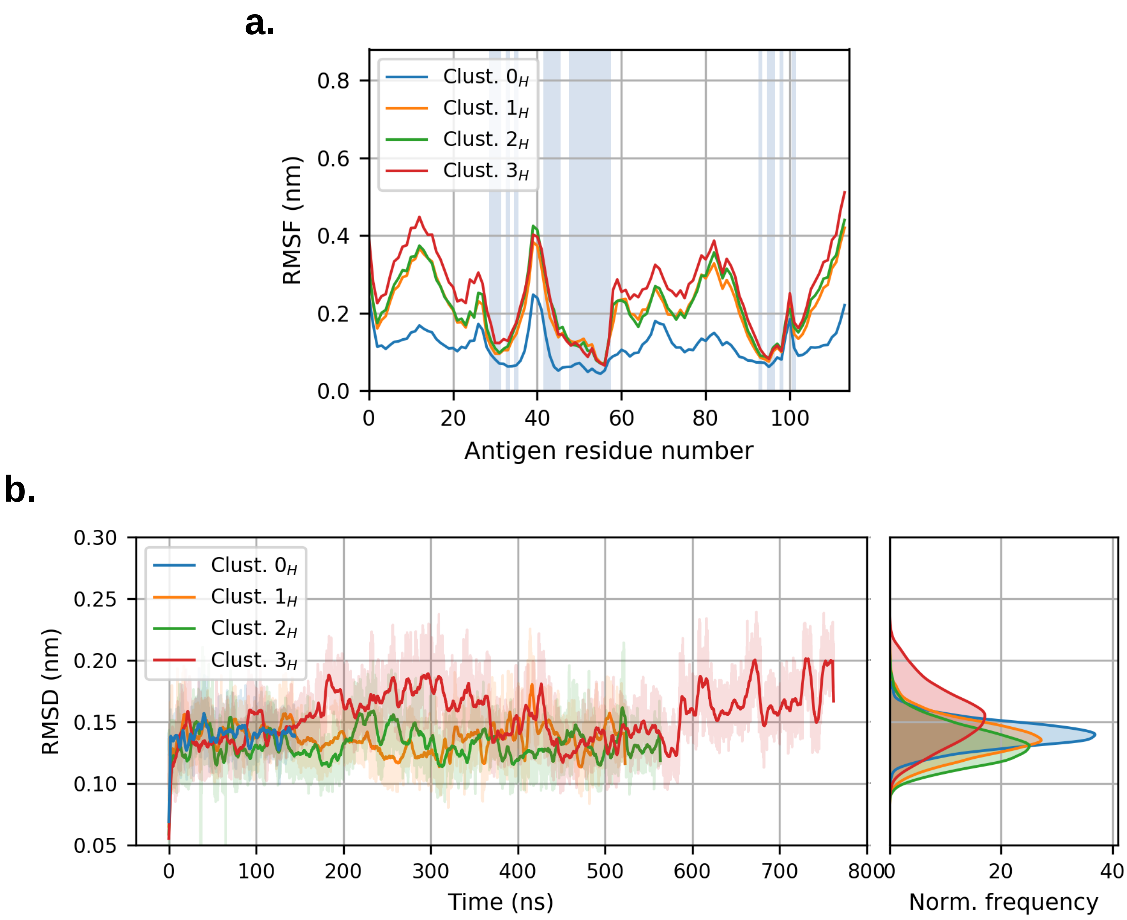

Figure S49: **a.** Root-mean-square fluctuations (RMSF) of C^{α} atoms of antigen PD-1, for each of the four clusters. The shaded areas correspond to residues of the epitope. **b.** Root-mean-square deviation (RMSD) of C^{α} atoms of PD-1 with respect to the starting conformation, and corresponding distributions.

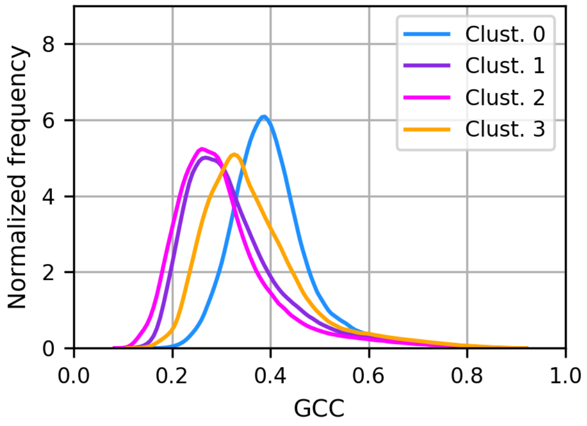

Figure S50: Distributions of the generalized correlation coefficient (normalized mutual information) within the antibody in the holo state, for each of the four clusters. The trend roughly follows the strength of the antibody-antigen binding.

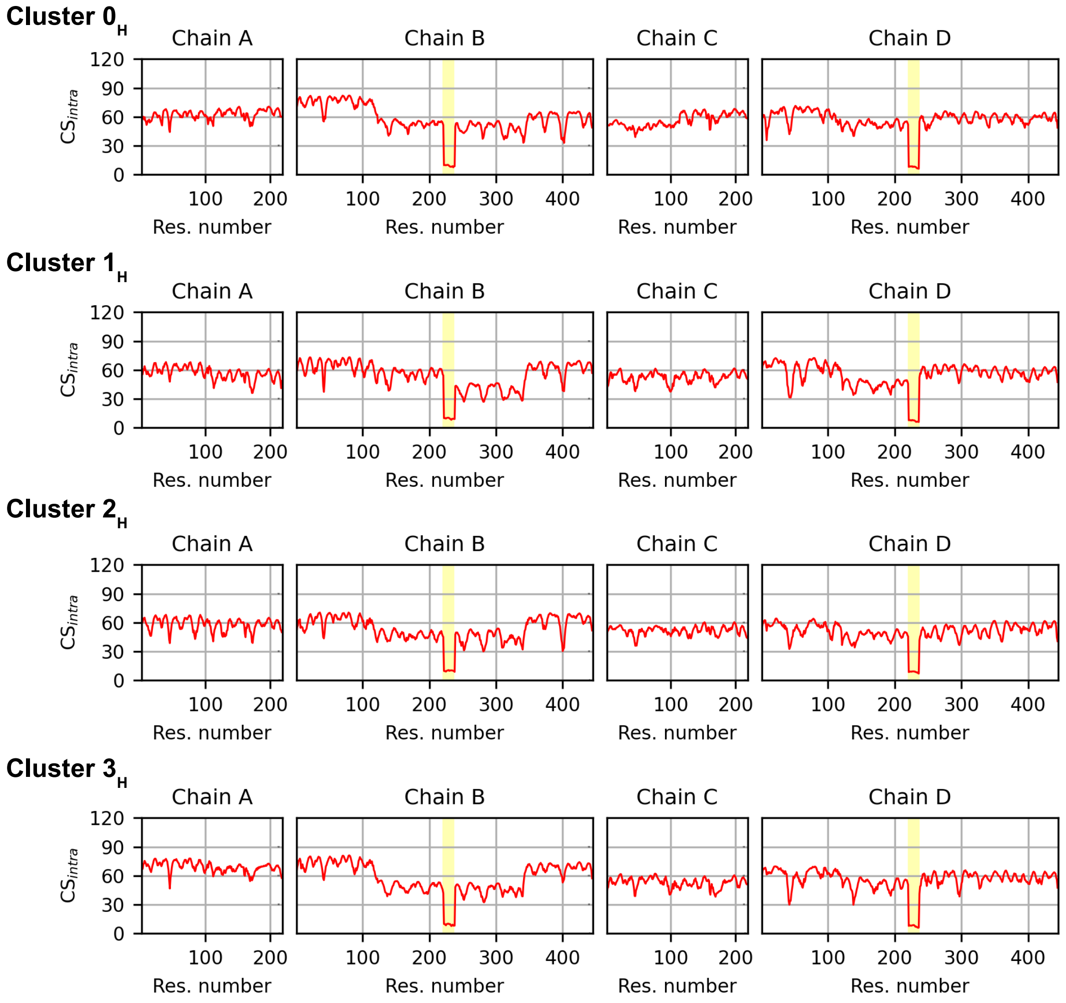

Figure S51: Intra-domain correlation score for each residue of the antibody in the holo state. The yellow-shaded areas represent the hinge regions.

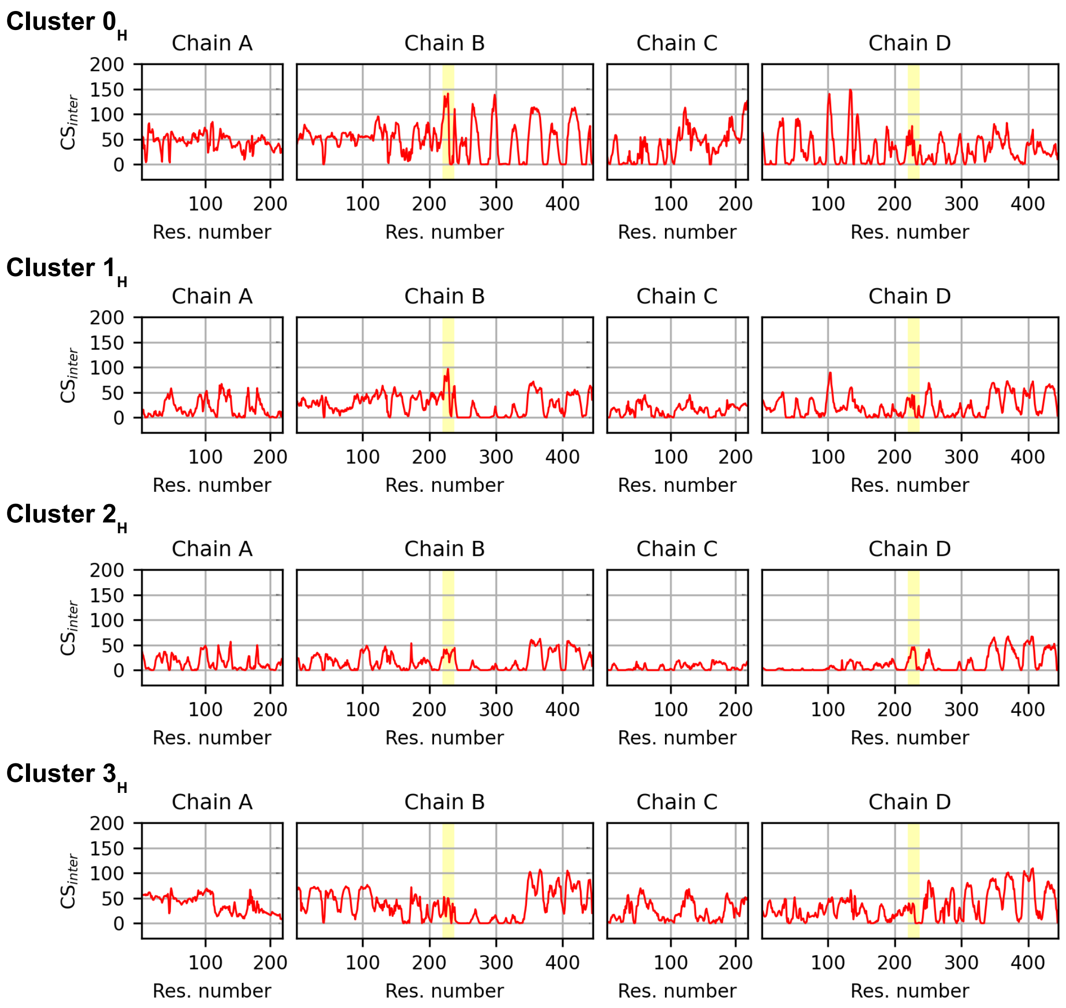

Figure S52: Inter-domain correlation coefficients for each residue of the antibody in the holo state. The yellow-shaded areas represent the hinge regions. In order to facilitate the comparison, CS_{inter} values have been plotted after filtering out low-value correlations (GCC<0.4).

Block 15	Cluster 0_H	Cluster 1_H	Cluster 2_H	Cluster 3_H
Block 1	0.36	0.19	0.11	0.24
Block 2	0.26	0.19	0.11	0.15
Block 3	0.37	0.30	0.11	0.28
Block 4	0.33	0.32	0.13	0.21
Block 5	0.40	0.31	0.18	0.27
Block 6	0.26	0.15	0.07	0.13
Block 7	0.35	0.20	0.1	0.25
Block 8	0.38	0.27	0.21	0.24
Block 9	0.52	0.31	0.27	0.26
Block 10	0.51	0.36	0.24	0.31
Block 11	0.45	0.31	0.22	0.26
Block 12	0.36	0.22	0.17	0.25
Block 13	0.30	0.24	0.12	0.25
Block 14	0.35	0.22	0.11	0.24
Block 15	1.07	0.78	0.60	0.66

Figure S53: Mutual information values for the holo cluster, between antigen (block 15) and all the structural domains of the antibody. The coloured blocks represent the highest values of each pair: in red the values grater than 0.5, in light blue the only 2 inter-domain correlations that are comparable between cluster 0_H and any of the other clusters (1_H).

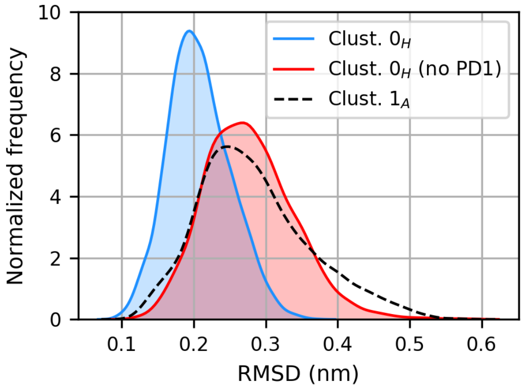

Figure S54: Distributions of the binding site RMSD values, in the holo cluster with the tightest binding and the strongest inter-protein correlations (namely cluster 0_H); in the structurally most similar apo cluster (cluster 1_A); and in the simulations started from the representative structure of 0_H after removal of the antigen.

S3 Additional tables

Table S2: Subdivision of the pembrolizumab/PD-1 complex into structural domains for the anaysis of correlations.

Chain	Domain ID	Residue range
A	1	1-112
	2	113-218
B	3	1-122
	4	123-218
	5	219-237
	6	238-340
	7	341-444
C	8	1-112
	9	113-218
D	10	1-122
	11	123-218
	12	219-237
	13	238-340
	14	341-444
PD-1	15	1-114

Table S3: Residue-residue contacts characterizing the conformational clusters of the apo system. Subscripts correspond to the chain ID. The interactions have been identified with PyContact [9]

Cluster ID	Fab1-Fc	Fab2-Fc	Fab1-Fab2
0_A	THR_B^{138}-ILE_B^{332}, VAL_A^{114}-ILE_B^{377}, THR_A^{113}-MET_B^{428}, THR_A^{113}-ILE_B^{377}, SER_B^{139}-GLU_B^{333}, THR_B^{138}-GLU_B^{333}, VAL_A^{114}-ASP_B^{376}, THR_A^{113}-HIS_B^{429}, THR_A^{113}-ASP_B^{376}, LYS_A^{111}-GLU_B^{380}, VAL_A^{114}-ALA_B^{378}, ASN_A^{156}-THR_D^{335}, THR_A^{113}-GLU_B^{430}, THR_A^{113}-HIS_B^{435}, THR_A^{113}-ALA_B^{378}, ARG_A^{112}-MET_B^{428}, ASN_A^{156}-LYS_D^{334}	No contacts	No contacts
1_A	SER_B^{139}-THR_B^{335}, SER_B^{141}-ILE_B^{336}, SER_B^{139}-LYS_B^{334}, SER_B^{139}-GLU_B^{333}, SER_B^{141}-THR_B^{335}, GLU_B^{140}-THR_B^{335}, LEU_A^{205}-LYS_B^{340}, VAL_A^{209}-LYS_B^{340}, VAL_A^{209}-ALA_B^{339}, SER_B^{141}-LYS_B^{334}, SER_A^{207}-LYS_B^{340}, GLU_B^{140}-SER_B^{337}, THR_A^{113}-LEU_B^{251}	No contacts	ARG_B^{217}-ASP_C^{126}, LYS_B^{221}-CYS_C^{218}, ARG_B^{217}-LYS_C^{130}, ARG_B^{217}-LEU_C^{129}
2_A	THR_B^{138}-ILE_B^{332}, THR_B^{138}-GLU_B^{333}, SER_B^{139}-VAL_B^{240}, SER_B^{139}-SER_B^{239}, SER_B^{139}-GLU_B^{333}, SER_B^{139}-LYS_B^{334}, ARG_B^{136}-PRO_B^{238}, ARG_B^{136}-SER_B^{239}, GLU_B^{140}-LYS_B^{334}, ARG_B^{136}-ILE_B^{332}, ARG_B^{136}-LEU_B^{328}	No contacts	No contacts

3_A	THR_B^{138}-ILE_B^{332}, SER_B^{139}-VAL_B^{458}, SER_B^{139}-GLU_B^{333}, SER_B^{139}-LYS_B^{334}, THR_B^{138}-GLU_B^{333}, ARG_B^{136}-PRO_B^{238}, ARG_B^{136}-LEU_B^{328}, SER_B^{139}-ILE_B^{332}	GLU_C^{217}-ASN_B^{297}, ASN_C^{214}-ASN_B^{297}, GLU_C^{217}-SER_B^{298}	LYS_B^{221}-ASP_C^{126}
4_A	THR_B^{138}-ASP_B^{265}, SER_B^{139}-ASP_B^{265}	THR_D^{138}-GLN_D^{268}, SER_D^{194}-LEU_D^{328}, SER_D^{139}-GLU_D^{269}, ARG_D^{136}-GLU_D^{269}, SER_D^{139}-ASP_D^{270}, THR_D^{138}-GLU_D^{269}, SER_D^{137}-GLU_D^{269}	LYS_B^{221}-ASP_C^{126}
5_A	No contacts	SER_D^{193}-LEU_D^{328}, SER_D^{194}-LEU_D^{328}, SER_D^{137}-GLU_D^{269}, THR_D^{138}-GLU_D^{269}, SER_D^{139}-GLU_D^{269}, SER_D^{139}-PRO_D^{271}, ARG_D^{136}-GLU_D^{269}, SER_D^{139}-ASP_D^{270}, SER_D^{141}-LYS_D^{326}, THR_D^{138}-GLN_D^{268}, GLU_D^{149}-PRO_D^{271}, SER_D^{141}-GLY_D^{327}	ARG_B^{217}-ASP_C^{126}, CYS_A^{218}-ARG_D^{217}, GLU_A^{217}-ARG_D^{217}, ARG_B^{217}-LYS_C^{130}, LYS_B^{221}-ASP_C^{126}

Table S4: Residue-residue interactions characterizing the conformational clusters of the holo system. Subscripts correspond to the chain ID.

Cluster ID	Fab1-Fc	Fab2-Fc	Fab1-Fab2
0_H	GLU_A^{17}-ARG_B^{255}, ASN_B^{162}-PHE_B^{296}, THR_A^{113}-ASP_B^{249}, PRO_A^{15}-ARG_B^{255}, THR_A^{113}-PRO_B^{247}, LYS_B^{199}-GLN_B^{295}, ARG_A^{112}-LYS_B^{246}, LEU_B^{166}-PHE_B^{296}, THR_A^{113}-LYS_B^{248}, GLU_A^{17}-ILE_B^{253}, SER_A^{14}-ARG_B^{255}, GLY_A^{16}-ILE_B^{253}, ASP_A^{174}-LYS_B^{246}, LYS_B^{199}-ASP_B^{265}, SER_B^{168}-ARG_B^{301}, ARG_A^{112}-PRO_B^{247}, ALA_B^{165}-PHE_B^{296}	ARG_C^{215}-ASN_B^{297}, LYS_C^{194}-ASN_B^{297}, ASN_C^{214}-ASN_B^{297}, GLY_C^{216}-ASN_B^{297}, ASN_C^{214}-GLN_B^{268}, ASN_C^{214}-SER_B^{298}, LYS_C^{194}-GLU_B^{294}	ARG_B^{217}-ASP_C^{126}, LYS_B^{221}-CYS_C^{218}, ASP_B^{215}-LYS_C^{187}, SER_B^{163}-GLU_C^{191}
1_H	THR_B^{138}-ILE_B^{332}, SER_B^{139}-ILE_B^{332}, SER_B^{141}-PHE_B^{241}, SER_B^{139}-LYS_B^{334}, SER_B^{139}-GLU_B^{333}, SER_B^{141}-LYS_B^{334}	ARG_C^{215}-ASN_B^{297}, LYS_C^{194}-GLN_B^{268}, ASN_C^{214}-GLN_B^{268}, ASN_C^{214}-GLU_B^{269}, GLU_C^{191}-ASN_B^{297}, GLY_C^{216}-GLN_B^{268}	ARG_B^{217}-ARG_D^{217}
2_H	THR_B^{138}-ILE_B^{332}, SER_B^{139}-VAL_B^{240}, THR_B^{138}-GLU_B^{333}, SER_B^{139}-GLU_B^{333}, SER_B^{139}-LYS_B^{334}, SER_B^{139}-ILE_B^{332}, SER_B^{139}-SER_B^{239}	ARG_D^{136}-GLU_D^{269}, VAL_C^{195}-ASN_B^{297}	LYS_B^{221}-CYS_C^{218}
3_H	SER_B^{139}-VAL_B^{264}, SER_B^{194}-THR_B^{299}	ASN_C^{214}-GLU_B^{269}, LYS_C^{211}-GLU_D^{269}, LYS_C^{194}-GLU_B^{269}, ARG_C^{215}-GLU_B^{269}, THR_C^{210}-GLU_D^{269}, VAL_C^{209}-ASP_D^{270}, GLU_D^{140}-LYS_D^{326}, SER_D^{141}-LEU_D^{328}, SER_C^{212}-GLU_D^{269}	No contacts

Table S5: Edges with the highest betweenness in the apo clusters.

Cluster ID	Residue 1	Domain	Residue 2	Domain
Cluster 0_A	ARG 217	4	PRO 225	5
	LEU 196	11	TYR 222	12
	TYR 222	12	PRO 228	12
	PRO 225	5	GLU 219	11
	ASN 141	9	TYR 177	9
	CYS 134	4	PRO 228	12
	GLU 109	8	TYR 177	9
	ASN 141	9	THR 190	11
	LEU 148	11	SER 184	11
	CYS 203	4	ARG 217	4
Cluster 1_A	ARG 217	4	ASP 126	9
	GLN 128	9	LYS 150	11
	LYS 150	11	TYR 183	11
	THR 117	10	TYR 183	11
	CYS 203	4	ARG 217	4
	SER 194	4	THR 198	4
	LYS 340	6	PRO 374	7
	TYR 222	5	PRO 224	5
	ARG 217	4	LYS 130	9
	THR 91	10	LEU 177	11
Cluster 2_A	GLU 109	8	TYR 177	9
	ASN 141	9	TYR 177	9
	ASN 141	9	THR 190	11
	ARG 136	4	ILE 332	6
	ARG 217	11	SER 220	11
	SER 119	10	PHE 153	11
	VAL 282	6	LEU 398	7
	TYR 319	6	LYS 334	6
	VAL 205	11	HIS 207	11
	CYS 203	11	ARG 217	11
Cluster 3_A	TYR 319	6	LYS 334	6
	SER 141	4	LYS 334	6
	SER 220	4	CYS 226	5
	PRO 224	5	ALA 132	11
	LYS 221	4	PRO 224	5
	THR 142	4	THR 190	4
	CYS 226	5	GLU 219	11
	THR 91	10	LEU 177	11
	PHE 120	2	THR 190	4
	LEU 148	11	SER 184	11
Cluster 4_A	LYS 221	4	ASP 126	9
	LYS 317	6	TYR 319	6
	ASP 126	9	GLN 128	9
	ASN 141	9	TYR 177	9
	TYR 319	6	CYS 321	6
	CYS 321	6	ILE 332	6
	GLN 128	9	LYS 150	11
	LYS 107	8	TYR 177	9
	LYS 317	6	TYR 373	7
	GLY 237	5	SER 239	6

Cluster 5_A	GLU 219	4	LYS 221	4
	LYS 221	4	ASP 126	9
	ILE 332	5	PHE 234	12
	ASP 126	9	GLN 128	9
	GLN 128	9	LEU 148	11
	PRO 232	12	PHE 234	12
	ASN 141	9	TYR 177	9
	THR 91	10	LEU 177	11
	PRO 230	12	PRO 232	12
	LEU 145	4	GLU 219	4

Table S6: Edges with the highest betweenness in the holo clusters.

Cluster ID	Residue 1	Domain	Residue 2	Domain
Cluster 0_H	ARG 217	4	ASP 126	9
	TYR 90	8	LYS 107	8
	TYR 201	4	ARG 217	4
	LYS 107	8	ARG 146	9
	ASP 126	9	GLN 128	9
	LYS 107	8	GLU 169	9
	GLU 294	6	LYS 194	9
	GLN 128	9	LEU 148	11
	GLU 294	6	ARG 301	6
	LYS 317	6	GLY 402	7
Cluster 1_H	TYR 222	5	PRO 227	5
	PRO 227	5	GLU 219	11
	LEU 196	4	TYR 222	5
	TYR 319	6	LYS 334	6
	LYS 317	6	TYR 319	6
	LEU 145	11	GLU 219	11
	ASN 141	2	TYR 177	2
	SER 141	4	LYS 334	6
	LYS 317	6	LEU 398	7
	PHE 120	2	THR 142	4
Cluster 2_H	ASN 141	9	TYR 177	9
	GLU 109	8	TYR 177	9
	LYS 107	8	GLU 169	9
	LYS 317	6	TYR 319	6
	LYS 317	6	TYR 373	7
	LYS 221	4	CYS 218	9
	ASN 141	2	TYR 177	2
	THR 168	9	GLU 169	9
	PRO 123	9	CYS 218	9
	TYR 90	8	LYS 107	8
Cluster 3_H	LEU 196	4	TYR 222	5
	TYR 222	5	PRO 227	5
	LEU 235	5	GLY 237	5
	PRO 227	5	GLU 219	11
	TYR 90	8	LYS 107	8
	ASN 315	6	TYR 373	7
	LYS 211	9	GLU 269	13
	LEU 145	4	LEU 196	4
	ASN 141	2	TYR 177	2
	ASN 141	9	TYR 177	9

Table S7: Enthalpic contributions to the antibody-PD1 binding energy, with respect to the value from the simulation of the Fab alone in complex with PD-1. Given the physical approximations, the results should be interpreted at a qualitative level.

Cluster ID	$\Delta\Delta H$ (Kcal/mol)
0_H	-24.1 ± 0.7
1_H	-16.0 ± 0.7
2_H	-17.5 ± 0.8
3_H	-22.0 ± 0.7

References

[1] E. Wang, H. Sun, J. Wang, Z. Wang, H. Liu, J. Z. Zhang, and T. Hou, "End-point binding free energy calculation with mm/pbsa and mm/gbsa: strategies and applications in drug design," *Chemical reviews*, vol. 119, no. 16, pp. 9478–9508, 2019.

[2] R. Kumari, R. Kumar, O. S. D. D. Consortium, and A. Lynn, "g_mmpbsa: A gromacs tool for high-throughput mm-pbsa calculations," *Journal of chemical information and modeling*, vol. 54, no. 7, pp. 1951–1962, 2014.

[3] M. C. Melo, R. C. Bernardi, C. De La Fuente-nunez, and Z. Luthey-Schulten, "Generalized correlation-based dynamical network analysis: a new high-performance approach for identifying allosteric communications in molecular dynamics trajectories," *The Journal of Chemical Physics*, vol. 153, no. 13, p. 134104, 2020.

[4] W. Humphrey, A. Dalke, and K. Schulten, "VMD – Visual Molecular Dynamics," *Journal of Molecular Graphics*, vol. 14, pp. 33–38, 1996.

[5] G. Tomasello, I. Armenia, and G. Molla, "The protein imager: a full-featured online molecular viewer interface with server-side hq-rendering capabilities," *Bioinformatics*, vol. 36, no. 9, pp. 2909–2911, 2020.

[6] G. Scapin, X. Yang, W. W. Prosise, M. McCoy, P. Reichert, J. M. Johnston, R. S. Kashi, and C. Strickland, "Structure of full-length human anti-pd1 therapeutic igg4 antibody pembrolizumab," *Nature Structural & Molecular Biology*, vol. 22, no. 12, pp. 953–958, 2015.

[7] R. R. Sokal and C. D. Michener, "A statistical method for evaluating systematic relationships.," *Univ. Kans. Sci. Bull.*, vol. 28, pp. 1409–1438, 1958.

[8] J.-P. Silva, O. Vetterlein, J. Jose, S. Peters, and H. Kirby, "The s228p mutation prevents in vivo and in vitro igg4 fab-arm exchange as demonstrated using a combination of novel quantitative immunoassays and physiological matrix preparation," *Journal of Biological Chemistry*, vol. 290, no. 9, pp. 5462–5469, 2015.

[9] M. Scheurer, P. Rodenkirch, M. Siggel, R. C. Bernardi, K. Schulten, E. Tajkhorshid, and T. Rudack, "Pycontact: Rapid, customizable, and visual analysis of noncovalent interactions in md simulations," *Biophysical journal*, vol. 114, no. 3, pp. 577–583, 2018.
